# Supplementary material for: Continuum and discrete modeling of binding-site distribution-mediated reactions on lipid surfaces
Source: Biophys J. 2026 Mar 10;125(7):1840–55. doi: 10.1016/j.bpj.2026.03.015 (PMC13181576; doi:10.1016/j.bpj.2026.03.015)
Supplement: Document S1. Figures S1–S7 [file mmc1.pdf]

Biophysical Journal, Volume *125*

Supplemental information

Continuum and discrete modeling of binding-site distribution-mediated reactions on lipid surfaces

Han Cao, Anirban Sen Gupta, and Karin Leiderman

Continuum and discrete modeling of binding-site distribution-mediated reactions on lipid surface

Authors: Han Cao, Anirban Sen Gupta, Karin Leiderman

SUPPLEMENTAL INFORMATION

PDE numerical scheme convergence analysis

To solve the coupled bulk–surface PDE model, we employed an operator-splitting time-stepping scheme. Here, we provide a simple convergence analysis to assess the numerical behavior of this scheme.

Because the full model involves multiple coupled species and nonlinear reaction terms and does not admit an analytical solution, we instead considered a reduced test problem that captures the essential bulk–surface coupling mechanism: a single chemical species undergoing diffusion in the bulk and reversible binding on the surface.

Specifically, we consider the following system:

$$\frac{\partial u}{\partial t} = D\Delta u, \tag{1}$$

$$\frac{\partial u^{\mathrm{m}}}{\partial t} = D^{m}\Delta_{\Gamma}u^{\mathrm{m}} + k_{\mathrm{on}}\,u\,(b_{\mathrm{total}} - u^{\mathrm{m}}) - k_{\mathrm{off}}\,u^{\mathrm{m}}, \tag{2}$$

$$D\,\frac{\partial u}{\partial \mathbf{n}} = k_{\mathrm{off}}\,u^{\mathrm{m}} - k_{\mathrm{on}}\,u\,(b_{\mathrm{total}} - u^{\mathrm{m}}), \tag{3}$$

where u denotes the solution-phase concentration and u^{m} denotes the surface-bound density. Here, b_{total} represents the total binding-site density on the surface, which is assumed to be spatially uniform. This configuration corresponds to binding patch distribution Pattern 1 shown in Fig. 4A of the main text, in which binding sites are uniformly distributed along the membrane.

Temporal convergence

We first examined the temporal convergence behavior of the operator-splitting scheme by systematically refining the time step size dt while keeping the spatial discretization fixed. For each choice of dt, we computed the spatially averaged bulk concentration $u(t)$, the spatially averaged surface-bound density $u^{\mathrm{m}}(t)$, and the total mass $u(t) + u^{\mathrm{m}}(t)$ with appropriate dimensional scaling. All quantities were normalized by their initial (or maximal) values.

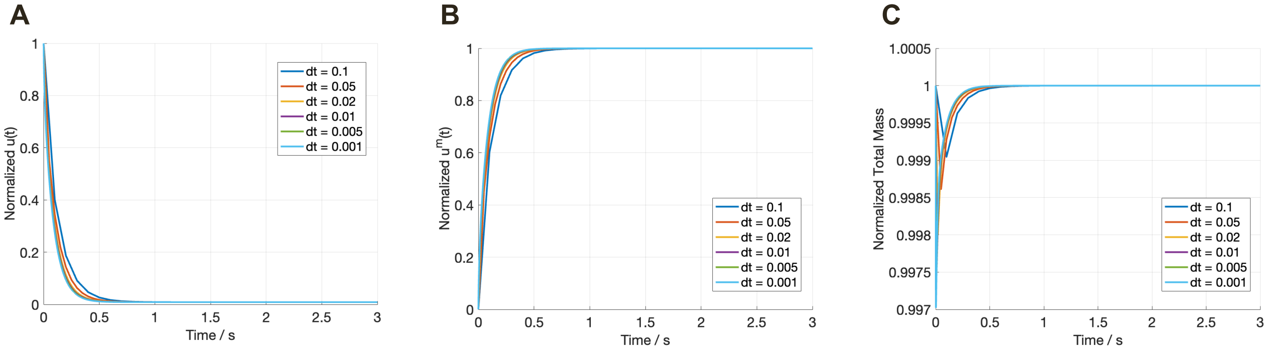

Figure S1: Temporal convergence of the operator-splitting scheme for the reduced bulk–surface binding model. (A) Normalized spatially averaged bulk concentration $u(t)$. (B) Normalized spatially averaged surface-bound concentration $u^{\mathrm{m}}(t)$. (C) Normalized total mass $u(t) + u^{\mathrm{m}}(t)$, with proper dimensional scaling. Results are shown for decreasing time step sizes dt.

Figure S1 shows the resulting time courses for decreasing values of dt. Panel A displays the normalized bulk concentration $u(t)$, Panel B shows the normalized surface-bound concentration $u^{\mathrm{m}}(t)$, and Panel C shows the normalized total mass. As the time step is refined, the solution trajectories converge toward a common curve for both the bulk and surface concentrations, indicating consistent temporal convergence of the scheme.

A small transient deviation from perfect mass conservation is observed at early times. This deviation is a numerical artifact of the operator-splitting treatment of the bulk–surface coupling. Because diffusion in the bulk and binding/unbinding reactions

on the surface are advanced sequentially, the bulk–surface mass exchange is not enforced in a fully coupled manner within each time step. As a result, small splitting errors in the total mass can be observed at early times. These deviations rapidly diminish as the simulation proceeds, and the total mass approaches a near-constant value at longer times.

Spatial convergence

We next examined the sensitivity of the numerical solution to the spatial discretization in the membrane-normal direction by refining the grid spacing dy, while keeping the time step fixed. Because the system is homogeneous in the x-direction for this test problem, only refinement in the y-direction was considered. Figure S2 shows the normalized solution-phase concentration u(t), surface-bound density $u^{\mathrm{m}}(t)$, and the normalized total mass for several values of dy.

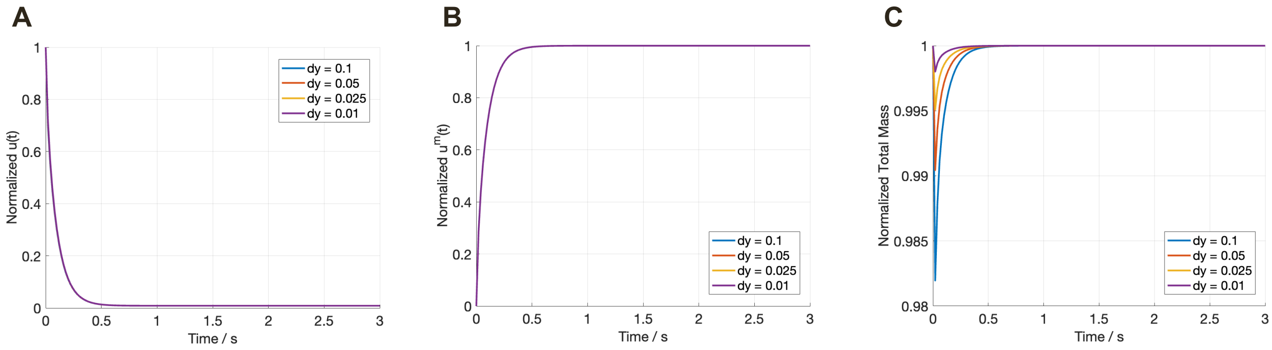

Figure S2: Spatial convergence of the operator-splitting scheme for the reduced bulk–surface binding model. (A) Normalized spatially averaged bulk concentration $u(t)$. (B) Normalized spatially averaged surface-bound concentration $u^{\mathrm{m}}(t)$. (C) Normalized total mass $u(t) + u^{\mathrm{m}}(t)$, with proper dimensional scaling. Results are shown for decreasing decreasing spatial grid spacing dy.

Across all tested spatial resolutions, the solution-phase and surface-bound concentration curves are nearly indistinguishable. This indicates that the temporal evolution of both u(t) and $u^{\mathrm{m}}(t)$ is well resolved even on relatively coarse spatial grids, and that further refinement in dy does not lead to visually discernible changes in these quantities.

In contrast, the small numerical artifact observed in the total mass conservation becomes progressively smaller as dy is refined. This behavior is consistent with discretization error in the bulk–surface coupling at the membrane boundary. As the spatial resolution increases, the discrete approximation of the normal flux and surface reaction terms improves, leading to better mass balance between the bulk and membrane compartments.

Temporal convergence of the full PDE model

We next examined the temporal convergence behavior of the full PDE model presented in the main text by repeating simulations using multiple time step sizes ($dt = 0.01$, 0.005, and 0.0025 s). Binding patch pattern was again set to be Pattern 1 shown in Fig. 4A from the main text.

As shown in Fig. S3, the temporal evolution of the solution-phase enzyme concentration $\mathrm{E}_1(t)$ and the membrane-bound enzyme density $\mathrm{E}_1^{\mathrm{m}}(t)$ is nearly identical across all tested values of dt, including both the peak magnitude and the overall time course. Here, $\mathrm{E}_1(t)$ denotes the spatially averaged enzyme concentration over the solution domain, and $\mathrm{E}_1^{\mathrm{m}}(t)$ denotes the spatially averaged enzyme density on the membrane.

These results indicate that, over the range of time step sizes considered, the numerical solution of the full PDE model exhibits weak sensitivity to temporal discretization, and that the time step used in the main simulations is sufficient to resolve the relevant dynamics.

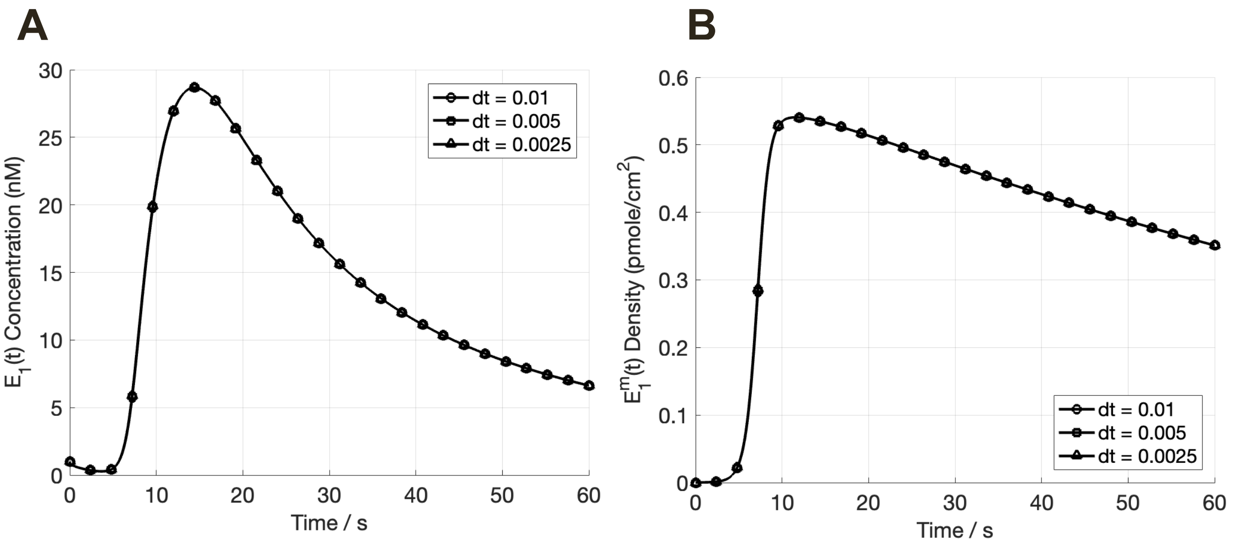

Figure S3: Temporal convergence behavior of the full PDE model presented in the main text. Results are shown for dt = 0.01, 0.005, and 0.0025 s. (A) Spatially averaged solution-phase enzyme concentration $E_1(t)$. (B) Spatially averaged membrane-bound enzyme density $E_1^m(t)$.

Taken together, these results demonstrate that the operator-splitting scheme employed here yields stable and convergent solution trajectories for the quantities of interest. Although the splitting approach introduces small transient mass-balance errors due to the sequential treatment of bulk diffusion and surface reactions, these artifacts are controlled and do not materially influence the qualitative or quantitative conclusions drawn from the simulations.

Vesicle geometry has little effect on product generation in 3D particle-based simulations

To enable precise control over binding patch geometry, we approximate the synthetic platelet as a cube, as Smoldyn currently provides more flexibility in defining surface regions on polyhedral geometries than on curved ones. Moreover, defining patches using triangular panels on a triangulated sphere introduces challenges: discretization results in panels of varying size and shape, making it difficult to uniformly control patch dimensions and spatial distribution.

To test the impact of synthetic platelet geometry, we performed simulations using three geometries with different levels of discretization: a perfect sphere, a cube, and a triangulated sphere composed of 80 triangular panels (see Fig. S4). The same reaction system and parameter values as described in the main study were used; however, in this analysis, binding site particles were allowed to diffuse freely across the entire platelet surface. If geometry played a significant role, such a setup would amplify any differences. Our results in Fig. S5, however, showed negligible differences in reaction outcomes across the three geometries, suggesting that platelet geometry has minimal influence under these conditions.

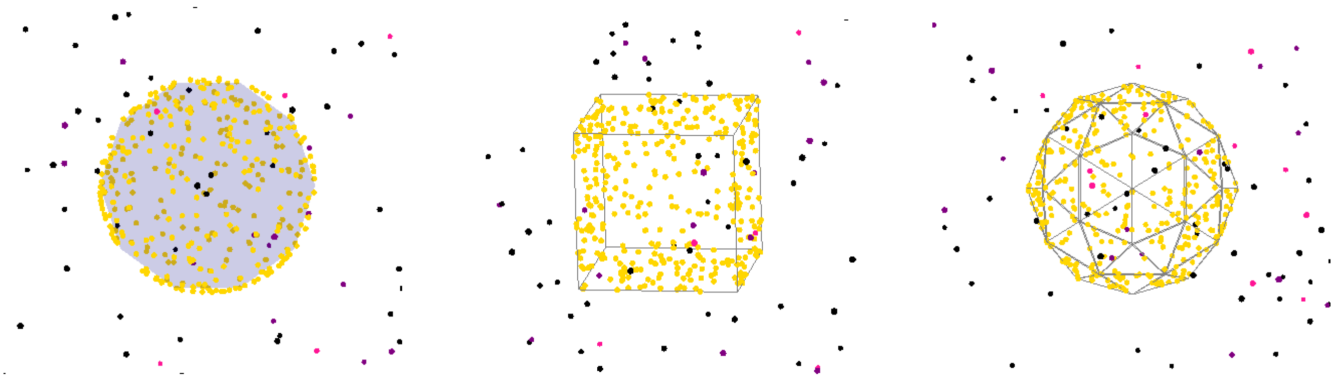

Figure S4: Snapshots of vesicles with different surface geometries. Yellow particles on the platelet surface represent binding site particles. (A) A vesicle with spherical surface. (B) A vesicle with cubic surface. (C) A vesicle with triangulated surface.

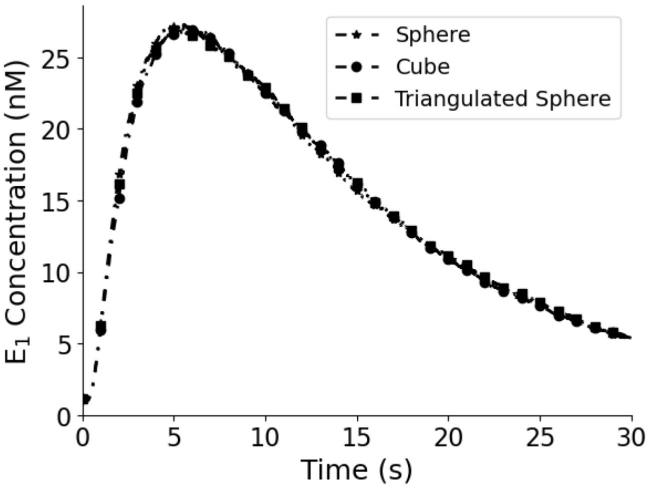

Figure S5: Enzyme generation curve for synthetic platelets of different geometries: a perfect sphere, a cube, and a triangulated sphere with 80 triangular panels.

Calculations

Platelet size and solution domain in PDE model

Assuming the surface of an activated platelet is a perfect sphere with radius

$$r_{\text{platelet}} = 2\mu\text{m},$$

then a cubic solution domain of side length

$$L_{\text{solution}} = 16\ \mu\text{m}$$

containing one platelet will yield a platelet concentration of

$$[\text{Platelet}] = 1/V_{\text{solution}} = 1/L^3_{\text{solution}} = 2.44 \times 10^5/\text{mm}^3,$$

which closely matches the physiological platelet concentration of approximately $2.5 \times 10^5/\text{mm}^3$. Placing the platelet at the center of the solution domain implies that the shortest distance from the platelet surface to the boundary of the solution domain is 6 μm, which is the value of $y_{\max}$ used in the PDE simulation (see Fig. S6).

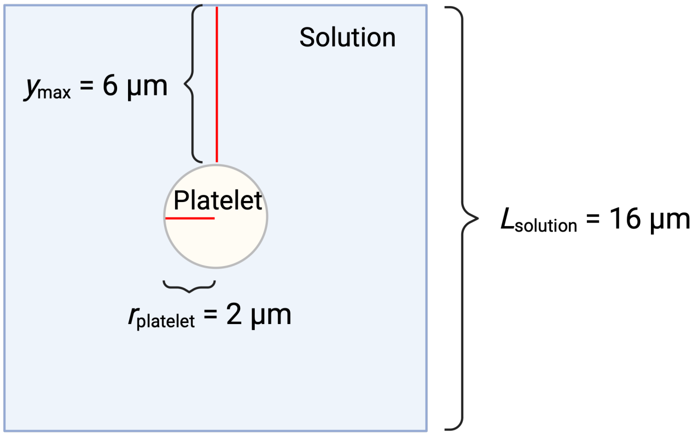

Figure S6: Schematic of a 2D cross-section of the platelet and its surrounding solution domain

Binding site density

We assumed each surface-bound protein occupies a circular area with a diameter of 5nm, giving a surface area of approximately

$$s_{\text{protein}} = (2.5\text{nm})^2\pi = 6.25\pi \text{ nm}^2.$$

This leads to an estimated maximum binding site density of $p_{\max} = 1/(6.25\pi\text{nm}^2) \approx 8.45 \text{ pmole/cm}^2$. In the PDE simulations, we used a slightly larger value of 10 pmole/cm^2.

Lipid vesicle size and domain in 3D particle-based model

We assumed the lipid vesicle has the radius of

$$r_{\text{PPN}} = 75 \text{ nm}$$

as in (1). Then, the surface area of the lipid vesicle is

$$s_{\text{PPN}} = 4\pi r_{\text{PPN}}^2 = 0.0225\pi \ \mu\text{m}^2.$$

If the lipid vesicle is approximated as a cube with the same total surface area, the corresponding side length is

$$l_{\text{PPN}} = \sqrt{s_{\text{PPN}}/6} \approx 0.10854 \ \mu\text{m}.$$

To determine the appropriate solution domain size for a particle-based simulation, we matched the surface area-to-volume ratio to that of a real platelet. A single physiological platelet has the same surface area as approximately 711 synthetic platelets:

$$\frac{s_{\text{platelet}}}{s_{\text{PPN}}} = \frac{4\pi r_{\text{platelet}}^2}{4\pi r_{\text{PPN}}^2} \approx 711.$$

Thus, to maintain equivalent surface area per unit volume, the solution domain for one synthetic platelet should be

$$v_{\text{solution}} = \frac{V_{\text{solution}}}{711},$$

yielding a cubic domain of side length

$$l_{\text{solution}} = \sqrt[3]{v_{\text{solution}}} \approx 1.8 \ \mu\text{m}.$$

Number of binding site particles in the particle-based model

Binding site particles

We assumed that 10% of the lipids on the synthetic platelet surface effectively contribute as binding sites. Accordingly, 10% of the synthetic platelet surface is designated as binding patches. To estimate the number of binding site particles per synthetic platelet, we divided the total area of the binding patches by the surface area occupied by a single protein:

$$\#\text{ of BS} = \frac{s_{\text{PPN}} \times 10\%}{s_{\text{protein}}} = 360.$$

Enzyme and zymogen particles

If the enzyme concentrations are set to $[E_1] = [E_2] = 1$ nM, then the number of E_1 and E_2 molecules in the simulation domain can be estimated by multiplying the concentration by the solution volume v_{solution}

$$\#\text{ of } E_1(E_2) = 1\text{ nM} \times v_{\text{solution}} \approx 3.51 \approx 4.$$

Since molecule counts must be integers in particle-based simulations, we initialize the system with 4 molecules each of E_1 and E_2 in the solution.

For the initial counts of zymogens, we use 400 molecules each of Z_1 and Z_2 in the solution.

Inhibitor particles

To ensure that all enzymes generated during the simulation can eventually be inhibited, we set the initial number of inhibitor molecules to 1000.

Stochastic effect of enzyme generation in 3D particle-based simulations

In some of our 3D particle-based simulations, enzyme inhibition occurs before amplification can begin, completely preventing the initiation of product generation—an inherently probabilistic outcome that cannot be captured by deterministic models.

In Fig.S7(A), we illustrate this effect using the case of a single binding patch as an example. Here, E_1 concentration trajectories from 200 simulation runs are shown, where the red line highlights runs in which no initiation occurs and the enzyme count decays to zero before product generation begins. Fig.S7(B) further summarizes the number of non-initiated simulations observed under each binding site distribution.

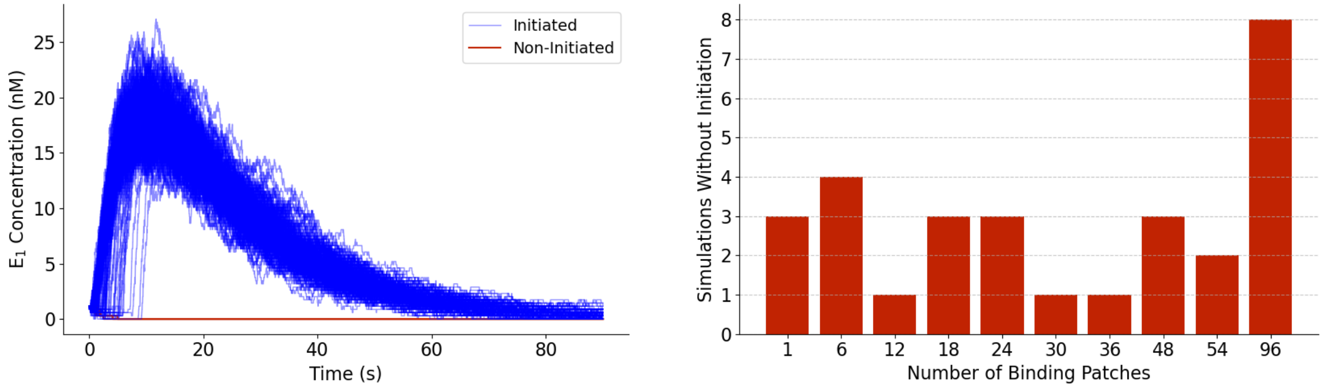

Figure S7: (A) Simulation trajectories for all runs with one binding patch. Trajectories where reactions were successfully initiated are shown in blue, while those with no reaction initiation are shown in red. This highlights the stochastic variability inherent at the molecular scale and low molecule counts, which deterministic models cannot capture. (B) Number of simulations with no reaction initiation (non-initiated) across different numbers of binding patches. The count reflects the stochastic failure of reaction initiation in particle-based simulations.

REFERENCES

1. Sekhon, U. D. S., K. Swingle, A. Girish, N. Luc, M. de la Fuente, J. Alvikas, S. Haldeman, A. Hassoune, K. Shah, Y. Kim, S. Eppell, J. Capadona, A. Shoffstall, M. D. Neal, W. Li, M. Nieman, and A. Sen Gupta, 2022. Platelet-Mimicking Procoagulant Nanoparticles Augment Hemostasis in Animal Models of Bleeding. *Science Translational Medicine* 14:eabb8975.
