# Supplementary material for: Continuum and discrete modeling of binding-site distribution-mediated reactions on lipid surfaces
Source: Biophys J. 2026 Mar 10;125(7):1840–55. doi: 10.1016/j.bpj.2026.03.015 (PMC13181576; doi:10.1016/j.bpj.2026.03.015)
Supplement: Document S2. Article plus supporting material [file mmc2.pdf]

Biophysical Journal
Article

Continuum and discrete modeling of binding-site distribution-mediated reactions on lipid surfaces

Han Cao,[1,2] Anirban Sen Gupta,[3] and Karin Leiderman[1,2,4,*]
[1]Department of Mathematics, University of North Carolina at Chapel Hill, Chapel Hill, North Carolina; [2]Computational Medicine Program, University of North Carolina at Chapel Hill, Chapel Hill, North Carolina; [3]Department of Biomedical Engineering, Case Western Reserve University, Cleveland, Ohio; and [4]Department of Biochemistry & Biophysics, University of North Carolina at Chapel Hill, Chapel Hill, North Carolina

ABSTRACT Cell surface-dependent biochemical reactions play a critical role in many biological processes. These include interactions between macromolecules in a three-dimensional bulk solution, macromolecules confined to a 2D membrane surface, and/or lipids that comprise the membrane. In blood coagulation, for instance, biochemical reactions that generate the key enzyme thrombin occur predominantly on the membrane surfaces of activated platelets. However, how the spatial distribution of the membrane binding sites affects enzymatic activity and reaction efficiency remains poorly understood. To bridge this gap, we employed both a partial differential equation model and a particle-based model to analyze a simplified biochemical reaction system in the presence of a lipid surface, inspired by coagulation reactions characterized by surface binding, inhibition, and positive feedback. Our analyses show that when binding sites are localized in a single patch, the optimal patch size reflects a trade-off between surface-bound reactant density and transport time to the surface. When binding sites are distributed among multiple patches, the partial differential equation model predicts that increasing the number of patches monotonically enhances efficiency. The particle-based model, which captures molecular-scale effects, reveals a nonmonotonic trend: efficiency increases with patch number initially and then declines as patches become overly fragmented. This discrepancy arises because continuum models allow for fractions of molecules to bind and interact, whereas biological reactions can only occur with whole molecules. Our results suggest that, for a fixed number of binding sites, moderately sized patches with high binding-site density are more efficient for enzyme generation compared with many small patches or few large patches of the same density. Our findings highlight the limitations of continuum models at molecular spatial scales, underscore the importance of discrete modeling in such regimes, and provide mechanistic insights regarding optimization of surface-dependent biomolecular reactions such as thrombin generation.

SIGNIFICANCE Many biochemical reactions take place on biological (e.g., cell) membranes, where macromolecules bind to lipid binding sites in the surface. The membrane not only provides these binding sites but also regulates the reactions through its structural properties. By confining molecular interactions to two dimensions instead of three, membranes enhance both the efficiency and regulation of these reactions. However, the influence of the spatial distribution of the lipid binding sites on reaction efficiency remains poorly understood. In this study, we used mathematical modeling and simulation to identify membrane binding patches of optimal size and binding-site density that maximize enzyme generation on biological membrane surfaces.

INTRODUCTION

Many biochemical reactions take place on biological surfaces, such as the cell membrane. During blood clotting, for example, activated platelet membranes expose phosphatidylserine-rich surfaces that support the assembly of coagulation enzyme-cofactor complexes (1,2), whereas subendothelial cells expose tissue factor that forms a catalytic complex with factor VIIa to initiate coagulation (3). In the immune system, red blood cells regulate complement activity at their surfaces through complement inhibitors (CD55, CD59) and receptors such as CR1 (4–7). Likewise, T cell activation

Submitted October 6, 2025, and accepted for publication March 5, 2026.
*Correspondence: karin.leiderman@unc.edu
Editor: N.G. Coganto phn.
https://doi.org/10.1016/j.bpj.2026.03.015
© 2026 The Authors. Published by Elsevier Inc. on behalf of Biophysical Society.
This is an open access article under the CC BY license (http://creativecommons.org/licenses/by/4.0/).

requires recognition of peptide-MHC complexes on antigen-presenting cell surfaces (8,9), and growth factors like EGF exert their effects by binding to membrane-bound receptors (10). These cases underscore that cell surfaces provide critical spatial organization and regulatory control for biochemical reactions. In this study, we are motivated by the biochemical reactions of blood coagulation that occur on activated platelet membranes during clotting, and by synthetic platelets, which were recently developed as biomimetic entities for hemostatic therapy (11).

Blood clotting is a tightly regulated physiological process that prevents blood loss after vascular injury (12). It involves the coordinated action of platelets and clotting factors, which together form a fibrin mesh that traps blood cells and seals the wound (13). Upon vessel injury, subendothelial matrix proteins become exposed and trigger platelet activation (14). Activated platelets adhere to the injury site and undergo aggregation, forming a loose platelet plug. Simultaneously, tissue factor (TF) expressed on subendothelial cells initiates coagulation, a complex network of enzymatic reactions and regulatory mechanisms involving multiple clotting factors and their inhibitors (15). A central component of this process is the procoagulant activity of platelets, which provides a lipid surface on which coagulation enzyme complexes form and promote efficient thrombin generation (16). Thrombin is a key enzyme that converts fibrinogen into fibrin, which polymerizes into a mesh that stabilizes the platelet plug (17).

Many of the essential enzymatic reactions in the coagulation cascade occur on the surface of activated platelets, which serves as a platform that localizes enzymatic activity and thereby enhances the efficiency of thrombin generation. The cascade is further characterized by strong positive feedback loops that amplify thrombin production, enabling a rapid and robust response to vascular injury. In parallel, inhibitory mechanisms tightly regulate the cascade to prevent excessive or inappropriate clot formation, modulating thrombin generation to avoid pathological thrombosis. For more detailed discussions on platelet activation, aggregation, and the biochemical mechanisms of coagulation, readers are referred to comprehensive reviews elsewhere (1,12,18,19).

Like all cellular membranes, the platelet membrane is primarily composed of various lipids, with phosphatidylserine (PS) being the most functionally significant during coagulation. These lipids exhibit an asymmetric distribution across the bilayer: in resting platelets, PS is enriched in the inner leaflet (20), but, upon activation, membrane inversion occurs, leading to the translocation of PS from the inner to the outer leaflet (21). PS exposure is a key event in rendering the platelet surface procoagulant, as it provides binding sites for coagulation factors such as VII, IX, X, and prothrombin (22). Certain clotting factors can associate with membrane binding sites formed by clusters of multiple PS lipids (Fig. 1 *A*). For instance, a cluster of six to eight PS molecules has been shown to serve as a functional binding site for factor X (23). After membrane binding, PS-interacting proteins can undergo lateral diffusion within the membrane that's rich in PS and interact with other membrane-bound factors (24). This facilitates the assembly of essential complexes, including tenase and prothrombinase, which are central to the coagulation cascade. We refer to these clusters of PS molecules as binding patches (Fig. 1 *B*). Accordingly, the spatial distribution of PS-rich binding patches may influence the assembly efficiency and spatial coordination of coagulation complexes, thereby affecting the overall kinetics of thrombin generation.

Previous modeling efforts

Previous mathematical modeling efforts of coagulation have recognized the important role of lipid binding sites. Fogelson and Kuharsky (25) focused on the surface of a single platelet and investigated a simplified enzyme system featuring surface-dependent reactions and positive feedback, modeled using partial differential equations (PDEs) in a one-dimensional domain. In their setup, one boundary represented the platelet surface that contains membrane binding sites. They showed that membrane binding site density acts as a biological switch: for the same enzyme level, changes in binding site density can shift the system from complete inhibition to robust product generation. Building on this, Beltrami and Jesty (26) explored how the size of membrane patches and flow serves as a threshold for feedback activation in a similar enzyme system using a PDE model. Their findings suggest that low flow rates or larger active membrane patches can exceed the activation threshold, whereas higher flow rates or

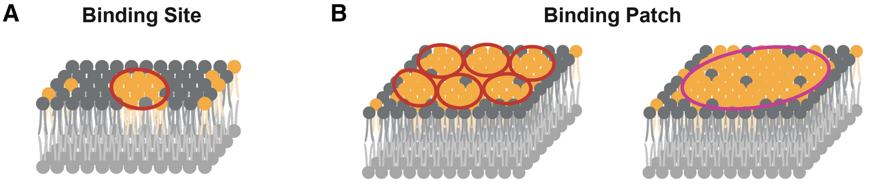

FIGURE 1 Illustration of coagulation factor binding sites and binding patches on the surface of activated platelets. Phosphatidylserine (PS) lipids are shown in yellow, and other lipids are shown in gray. (*A*) A cluster of PS molecules forms a single coagulation factor binding site (*indicated by the red circle*) on the platelet membrane. (*B*) Multiple binding sites (*small red circles, left*) form a larger PS patch (*large purple circle, right*), which facilitates lateral diffusion and interactions of membrane-bound coagulation factors.

smaller membrane patches can prevent initiation. Kuharsky and Fogelson later developed an ordinary differential equation (ODE) model that incorporated a more complex reaction system under flow conditions, focusing on the TF pathway (27). A key finding from their study was that, consistent with the earlier simplified system, the model exhibits a threshold-like response to changes in the availability of surface binding sites. Bungay and Gentry modeled lipid binding sites explicitly, within the framework of a static thrombin generation assay, which is in the absence of flow (28). They provided a comprehensive ODE model of lipid-mediated thrombin generation, where lipids were represented in the model by an effective concentration that reflects their surface availability. Their model showed that thrombin generation increased as lipid concentration increased, which through more recent studies, we now know should follow nonmonotonic behavior (29). Leiderman and Fogelson developed a spatiotemporal model that incorporates surface-mediated coagulation and describes clot formation under flow conditions, performing extensive investigations into platelet deposition and blood coagulation (30,31). In their work, the concentration of different types of platelets is tracked, and available binding sites on each platelet species are modeled using volume concentrations rather than explicit surface densities. Across these studies, membrane binding sites have typically been modeled as averaged surface densities or effective volume concentrations. Except for the work of Fogelson and Kuharsky (25), these models consider spatial scales far larger than that of a single platelet. Our study addresses a gap in understanding how the heterogeneous spatial organization of binding sites on individual membrane surfaces influences reaction dynamics.

In addition to coagulation-specific models, more studies have investigated the general question of how efficiently diffusing molecules can find and bind to receptors on a surface. The classical Berg-Purcell framework established the physical limit of this capture process (32), and later work extended the analysis to include the finite speed of receptor binding and unbinding (33). Lawley et al. further showed that the spatial arrangement of receptors matters in source detecting: evenly distributed receptors yield direction-independent sensing accuracy, whereas clustered receptors achieve higher accuracy when the source is aligned with the cluster (34). Other studies highlighted that binding events are highly variable when only a few molecules are present (35), and that the timing of early molecular arrivals already carries directional information (36). In these works, "efficiency" refers specifically to the binding step itself, but the underlying principles, such as diffusion limits, receptor distribution, and stochastic fluctuations, are broadly relevant to surface-mediated biochemical processes. Rather than focusing solely on surface binding, our work considers enzymatic reactions on the membrane, where reactants encounter each other and catalytic turnover introduces an additional layer of regulation beyond the initial transport.

Modeling spatially resolved reactions with molecular granularity

ODE and PDE models provide macroscopic, deterministic descriptions of chemical reactions by tracking concentrations or surface densities across space and time. However, they often fail to capture the stochastic and discrete behavior of individual molecules—especially when molecule counts are low or the spatial resolution of interest approaches molecular scales, as encountered in our study.

A widely used class of spatial stochastic models that balances spatial resolution and computational efficiency includes the reaction-diffusion master equation (RDME) (37,38) and associated algorithms such as the spatial Gillespie algorithm (39). These methods discretize space into subvolumes (voxels), simulate stochastic reactions within each voxel, and model diffusion as random hopping between neighboring voxels. Although they offer a balance between spatial resolution and computational efficiency, RDME-based approaches lack the ability to resolve individual molecular interactions in continuous space.

In contrast, particle-based methods incorporate molecular granularity more explicitly. These methods represent molecules as point particles diffusing in continuous space and time, with probabilistic reactions governed by interparticle distances and reaction radii. A prominent class of such approaches is the Smoluchowski-type model (40), which treats bimolecular reactions as diffusion-limited processes. Algorithms based on this framework have been implemented in simulators such as Smoldyn (41) and MCell (42), both of which enable efficient simulations of stochastic reaction-diffusion systems in complex geometries. Other notable methods include Green's function reaction dynamics (GFRD) (37), which uses analytical solutions (Green's functions) of the diffusion equation to compute the next reaction or interaction event. This allows for large jumps in time and space, making GFRD highly efficient in dilute systems. However, the method can become computationally expensive in dense systems due to frequent interactions and reduced event-prediction efficiency. For comprehensive discussions on model complexity, spatial resolution, and computational trade-offs, we refer readers to review articles such as (43–46).

Our goal was to investigate how the spatial distribution of binding sites on lipid vesicle surfaces influences reaction dynamics on those surfaces and in a surrounding solution. We also aimed to identify an optimal spatial arrangement of binding sites that maximizes product generation. We used the term "vesicle surface" as a general representation of various types of cellular surfaces. We employed a continuum PDE model and a discrete particle-based model to study a

simplified reaction system (25) that includes positive feedback and surface dependence. The PDE model was solved in a two-dimensional bulk domain with the membrane represented as a one-dimensional boundary. This reduction in dimensionality of numerical implementation was chosen for computational efficiency. Several limiting cases were examined, and different spatial distributions of binding sites were compared. We next performed a quasi-two-dimensional particle-based simulation to provide a qualitative comparison with the PDE predictions. Finally, a fully three-dimensional particle-based model was implemented to demonstrate the applicability of the approach in a realistic geometry. Our results showed that the spatial distribution of binding sites critically affects product generation. Considering a fixed number of binding sites, when catalytic activity on the membrane surface is limiting, binding sites clustered in one small dense patch accelerated product generation, whereas under transport limitation, a large, less dense patch was favorable. If the same number of binding sites were distributed into multiple patches, the continuum PDE model and discrete particle-based model had contrasting predictions about product generation. The continuum PDE model predicted that efficiency increases monotonically to saturation as the number of patches increased, even when patch sizes were smaller than a single protein. The particle-based model revealed a non-monotonic dependence of product generation on the number of patches, with highest efficiency at intermediate numbers of patches. This discrepancy between model predictions arises for high numbers of binding patches because the continuum model represents fractions of binding sites to which proteins cannot physically bind, resulting in an over-accumulation of bound proteins and, consequently, an overestimation of product generation.

MATERIALS AND METHODS

We created two reaction-diffusion models to simulate enzymatic reactions in solution and on membrane and to study the role of spatial distribution of surface binding sites; one model consists of PDEs, and one is particle based.

The reaction system for both models is based on a previously published model (25) describing two interacting zymogen-enzyme pairs (Z_1, E_1), (Z_2, E_2) with positive feedback. A schematic of these reactions is in Fig. 2. The enzymes and zymogens in solution can bind to available binding sites on the membrane surface and become surface-bound species Z_i^{m}, E_i^{m} ($i = 1{,}2$); surface-bound species can unbind from the surface, transitioning back into the solution. The catalytic reactions are assumed to occur only on the surface, where E_1^{m} activates Z_2^{m} into E_2^{m}, and E_2^{m} activates Z_1^{m} to E_1^{m}. Enzymes are irreversibly inhibited by the inhibitor In, both in the solution and on the surface. The binding to and unbinding from the surface reactions are described as follows:

$$E_1 + B^{avail} \underset{k_{1\mathrm{off}}}{\overset{k_{1\mathrm{on}}}{\rightleftharpoons}} E_1^{\mathrm{m}}, \qquad Z_1 + B^{avail} \underset{k_{1\mathrm{off}}}{\overset{k_{1\mathrm{on}}}{\rightleftharpoons}} Z_1^{\mathrm{m}},$$

$$E_2 + B^{avail} \underset{k_{2\mathrm{off}}}{\overset{k_{2\mathrm{on}}}{\rightleftharpoons}} E_2^{\mathrm{m}}, \qquad Z_2 + B^{avail} \underset{k_{2\mathrm{off}}}{\overset{k_{2\mathrm{on}}}{\rightleftharpoons}} Z_2^{\mathrm{m}},$$

Here, B^{avail} represents available binding sites. The enzymatic reactions on the surface are given by the following:

$$E_1^{\mathrm{m}} + Z_2^{\mathrm{m}} \underset{k_1^-}{\overset{k_1^+}{\rightleftharpoons}} \mathrm{C}_1 \overset{k_1^{\mathrm{cat}}}{\rightarrow} E_1^{\mathrm{m}} + E_2^{\mathrm{m}},$$

$$E_2^{\mathrm{m}} + Z_1^{\mathrm{m}} \underset{k_2^-}{\overset{k_2^+}{\rightleftharpoons}} \mathrm{C}_2 \overset{k_2^{\mathrm{cat}}}{\rightarrow} E_2^{\mathrm{m}} + E_1^{\mathrm{m}},$$

And, the inhibition reactions are as follows:

$$E_1 + In \overset{k_1^{\mathrm{in}}}{\rightarrow} E_1 : In, \qquad E_2 + In \overset{k_2^{\mathrm{in}}}{\rightarrow} E_2 : In,$$

$$E_1^{\mathrm{m}} + In \overset{k_1^{\mathrm{in}}}{\rightarrow} E_1^{\mathrm{m}} : In, \qquad E_2^{\mathrm{m}} + In \overset{k_2^{\mathrm{in}}}{\rightarrow} E_2^{\mathrm{m}} : In.$$

PDE model

The PDE model incorporates reaction terms, defined by the reaction scheme above, and diffusion terms. We implemented a two-dimensional PDE framework to describe the diffusion–reaction system, explicitly tracking species on the vesicle surface as well as in the surrounding solution. Model species' names are denoted with uppercase letters (e.g., E_1, E_1^{m}), with their concentrations and surface densities denoted with lowercase variables (e.g., e_1, e_1^{m}). Solution-phase species have units of concentration, and surface-bound species have units of surface density. The solution-phase equations are written as follows:

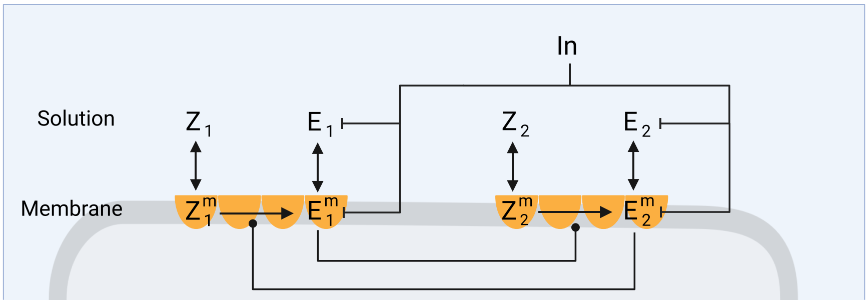

FIGURE 2 Schematic of the cell surface-dependent reaction system that includes a positive feedback loop and inhibition. Two enzyme-zymogen pairs (E_1, Z_1) and (E_2, Z_2) are considered. Both enzymes and zymogens can reversibly bind to available surface binding sites (*represented by orange hemispheres*) to form surface-bound species ($\mathrm{E}_i^{\mathrm{m}}$, $\mathrm{Z}_i^{\mathrm{m}}$, $i = 1{,}2$, m represents membrane-bound), or unbind to return to the solution phase (E_i, Z_i). On the membrane surface, $\mathrm{E}_1^{\mathrm{m}}$ activates $\mathrm{Z}_2^{\mathrm{m}}$ into $\mathrm{E}_2^{\mathrm{m}}$, and $\mathrm{E}_2^{\mathrm{m}}$ activates $\mathrm{Z}_1^{\mathrm{m}}$ into $\mathrm{E}_1^{\mathrm{m}}$, establishing a positive feedback loop. An inhibitor species (In) irreversibly inhibits both E_1 and E_2, in solution and on the membrane. In the schematic, conversion reactions are represented by pointed arrowheads, enzymatic reactions by oval-headed arrows, and inhibitory interactions by bar-headed lines.

$$\frac{\partial e_1}{\partial t} = D_1 \Delta e_1 - k_{1\text{in}} e_1, \tag{1}$$

$$\frac{\partial z_1}{\partial t} = D_1 \Delta z_1, \tag{2}$$

$$\frac{\partial e_2}{\partial t} = D_2 \Delta e_2 - k_{2\text{in}} e_2, \tag{3}$$

$$\frac{\partial z_2}{\partial t} = D_2 \Delta z_2. \tag{4}$$

For simplicity, we did not explicitly model the inhibitor. Instead, its effect was approximated by first-order decay terms like $-k_{1\text{in}}e_1$ in Eq. 1. Equations on the membrane surface are given by the following:

$$\frac{\partial e_1^{\text{m}}}{\partial t} = D_1^{\text{m}} \Delta_\Gamma e_1^{\text{m}} + k_{1\text{on}} e_1 b^{\text{avail}} - k_{1\text{off}} e_1^{\text{m}} - k_1^+ e_1^{\text{m}} z_2^{\text{m}} + k_1^- c_1 + k_1^{\text{cat}} c_1 + k_2^{\text{cat}} c_2 - k_{1\text{in}}^{\text{m}} e_1^{\text{m}}, \tag{5}$$

$$\frac{\partial z_1^{\text{m}}}{\partial t} = D_1^{\text{m}} \Delta_\Gamma z_1^{\text{m}} + k_{1\text{on}} z_1 b^{\text{avail}} - k_{1\text{off}} z_1^{\text{m}} - k_2^+ e_2^{\text{m}} z_1^{\text{m}} + k_2^- c_2, \tag{6}$$

$$\frac{\partial e_2^{\text{m}}}{\partial t} = D_2^{\text{m}} \Delta_\Gamma e_2^{\text{m}} + k_{2\text{on}} e_2 b^{\text{avail}} - k_{2\text{off}} e_2^{\text{m}} - k_2^+ e_2^{\text{m}} z_1^{\text{m}} + k_2^- c_2 + k_1^{\text{cat}} c_1 + k_2^{\text{cat}} c_2 - k_{2\text{in}}^{\text{m}} e_2^{\text{m}}, \tag{7}$$

$$\frac{\partial z_2^{\text{m}}}{\partial t} = D_2^{\text{m}} \Delta_\Gamma z_2^{\text{m}} + k_{2\text{on}} z_2 b^{\text{avail}} - k_{2\text{off}} z_2^{\text{m}} - k_1^+ e_1^{\text{m}} z_2^{\text{m}} + k_1^- c_1, \tag{8}$$

$$\frac{\partial c_1}{\partial t} = D_{\text{c}}^{\text{m}} \Delta_\Gamma c_1 + k_1^+ e_1^{\text{m}} z_2^{\text{m}} - k_1^- c_1 - k_1^{\text{cat}} c_1, \tag{9}$$

$$\frac{\partial c_2}{\partial t} = D_{\text{c}}^{\text{m}} \Delta_\Gamma c_2 + k_1^+ e_2^{\text{m}} z_1^{\text{m}} - k_2^- c_2 - k_2^{\text{cat}} c_2, \tag{10}$$

$$b^{\text{avail}} = b - e_1^{\text{m}} - e_2^{\text{m}} - z_1^{\text{m}} - z_2^{\text{m}} - 2c_1 - 2c_2. \tag{11}$$

Notice that the Laplacian operator Δ acts on the bulk solution domain, whereas the surface Laplacian Δ_Γ acts on the membrane domain. The superscript m in the diffusion coefficients represents mobility in the membrane surface and distinguishes these coefficients from their solution-phase counterparts. We now provide explanations of terms in Eq. 5 to help readers build intuition for the equations. The term $D_1^{\text{m}} \Delta_\Gamma e_1^{\text{m}}$ represents random diffusion. The term $k_{1\text{on}} e_1 b^{\text{avail}}$ corresponds to the rate at which solution-phase enzymes bind to available binding sites on the vesicle surface. At each spatial location, b^{avail} denotes the density of available binding sites, calculated by subtracting the density of surface-bound proteins from the local total binding-site density b, as defined in Eq. 11. The total density b is constant within binding patches and zero elsewhere on the surface. The factor of 2 in front of c_1 and c_2 reflects that each surface complex comprises two proteins and therefore occupies twice the number of binding sites compared with a single protein. The term $k_{1\text{off}} e_1^{\text{m}}$ represents the rate at which E_1 dissociates from the binding sites. The terms $-k_1^+ e_1^{\text{m}} z_2^{\text{m}} + k_1^- c_1$ describe the association between membrane-phase enzymes and the corresponding zymogens to form a complex, as well as the inverse process, where the complex dissociates. The terms $+k_1^{\text{cat}} c_1 + k_2^{\text{cat}} c_2$ capture the enzymatic activation of the zymogens into their corresponding enzymes. Lastly, $-k_{1\text{in}}^{\text{m}} e_1^{\text{m}}$ represents the inhibition of the enzymes on the membrane surface.

The exchange of species between the solution and the membrane surface imposes Robin boundary conditions on the solution-phase equations, given by the following expressions:

$$D_1 \frac{\partial e_1}{\partial \mathbf{n}} = k_{1\text{off}} e_1^{\text{m}} - k_{1\text{on}} e_1 b^{\text{avail}}, \tag{12}$$

$$D_1 \frac{\partial z_1}{\partial \mathbf{n}} = k_{1\text{off}} z_1^{\text{m}} - k_{1\text{on}} z_1 b^{\text{avail}}, \tag{13}$$

$$D_2 \frac{\partial e_2}{\partial \mathbf{n}} = k_{2\text{off}} e_2^{\text{m}} - k_{2\text{on}} e_2 b^{\text{avail}}, \tag{14}$$

$$D_2 \frac{\partial z_2}{\partial \mathbf{n}} = k_{2\text{off}} z_2^{\text{m}} - k_{2\text{on}} z_2 b^{\text{avail}}, \tag{15}$$

$$b^{\text{avail}} = b - e_1^{\text{m}} - e_2^{\text{m}} - z_1^{\text{m}} - z_2^{\text{m}} - 2c_1 - 2c_2,$$

Here, $\mathbf{n}$ denotes the outward unit normal vector at the membrane surface, pointing from the solution domain toward the membrane. On the remaining portions of the vesicle surface where binding is not allowed, we impose no-flux boundary conditions:

$$\frac{\partial e_1}{\partial \mathbf{n}} = \frac{\partial z_1}{\partial \mathbf{n}} = \frac{\partial e_2}{\partial \mathbf{n}} = \frac{\partial z_2}{\partial \mathbf{n}} = 0. \tag{16}$$

Detailed parameter information and default values are given in Table 1.

The initial conditions for both enzyme-zymogen pairs are also chosen symmetrically: $e_1(0, x, y) = e_2(0, x, y) = 1$ nM, $z_1(0, x, y) = z_2(0, x, y) = 1000$ nM, and initially, there are no chemicals on the membrane, so $e_1^{\text{m}}(0,x) = e_2^{\text{m}}(0,x) = z_1^{\text{m}}(0,x) = z_2^{\text{m}}(0,x) = 0$ pmole/cm^2.

Spatial domain

Instead of solving a fully three-dimensional PDE system, we numerically implemented the model on a two-dimensional computational domain as a reduced representation. This approach enables efficient exploration of how binding-site distribution influences reaction outcomes while preserving the key transport and reaction mechanisms of interest. For the spatial domain, we focused on a small patch of the membrane surface and its adjacent solution, which is a zoomed-in view of a single vesicle embedded in solution with many other vesicles. A schematic of this is depicted in Fig. 3 *A–C*. The vesicles in this scenario could represent cells such as platelets or synthetic platelet-mimetic hemostatic particles (11) where coagulation reactions occur on lipid patches on the surface. For simplicity, we ignore the membrane curvature. This results in the two-dimensional computational domain $\Omega = [0, x_{\max}] \times [0, y_{\max}]$, as shown in Fig. 3 *C*. The one-dimensional membrane surface lies along $y = 0$, where binding patches are distributed. These patches are illustrated as yellow line segments, in contrast to the gray segments that represent the rest of the vesicle surface.

The parameter $x_{\max}$ defines the length of the membrane segment in the domain. A vesicle radius of 2 μm is adopted, consistent with the characteristic size of platelets, so that $x_{\max} = 12$ μm provides an approximation of the two-dimensional circumference. The parameter $y_{\max}$ represents half the average shortest distance between adjacent vesicle membranes. A large $y_{\max}$ corresponds to a low vesicle concentration, whereas a small value

TABLE 1 Parameters

Parameter	Description	Value	Unit	Note
$D_i \quad (i = 1,2)$	diffusion coefficients of species in the solution	$5 \cdot 10^{-8}$	cm^2/s	a
$D_i^{\mathrm{m}} \quad (i = 1,2,c)$	diffusion coefficients of species on the membrane	$5 \cdot 10^{-9}$	cm^2/s	a
$k_{i\ \mathrm{in}}, k_{i\ \mathrm{in}}^{\mathrm{m}} \quad (i = 1,2)$	rate constants for the inhibition of enzymes	0.01	$1/\mathrm{s}$	b
$k_{i\ \mathrm{on}} \quad (i = 1,2)$	association rate constants for surface binding	0.01	$1/(\mathrm{nM} \cdot \mathrm{s})$	c
$k_{i\ \mathrm{off}} \quad (i = 1,2)$	dissociation rate constants of surface-bound molecules from membrane	0.1	$1/\mathrm{s}$	c
$k_i^{+} \quad (i = 1,2)$	association rate constants between membrane-bound enzyme and zymogen	0.3	$1/(\mathrm{pmole}/\mathrm{cm}^2 \cdot \mathrm{s})$	d
$k_i^{-} \quad (i = 1,2)$	dissociation rate constants of surface complex	1	$1/\mathrm{s}$	d
$k_i^{\mathrm{cat}} \quad (i = 1,2)$	rate constants for enzyme activation	30	$1/\mathrm{s}$	e
$b_{\max}$	upper bound of binding site density on membrane	10	$\mathrm{pmole}/\mathrm{cm}^2$	f

[a]The diffusion coefficients of solution-phase chemical species are taken from (47), but they are reduced by one order of magnitude to enhance the transport effect. The lateral diffusion coefficients on the membrane are taken to be two orders of magnitude smaller than those in the solution (48).
[b]The second-order inhibition rate of thrombin by antithrombin is $1.4 \times 10^{-5}\ \mathrm{nM}^{-1}\mathrm{s}^{-1}$ (49). Given that the plasma concentration of antithrombin is approximately 2300 nM (50), the effective first-order inhibition rate is estimated to be a product of the second-order rate and antithrombin concentration. This yields a value on the order of $0.01\ \mathrm{s}^{-1}$.
[c]We assumed the same rates as in the Fogelson-Kuharsky study (25).
[d]We chose the association rate constant to be $0.3\ 1/(\mathrm{pmole}/\mathrm{cm}^2 \cdot \mathrm{s})$, which represents fast association yet allows for exploration of different limiting regime.
[e]We assumed the activation rate of prothrombin by prothrombinase is $30\ \mathrm{s}^{-1}$ (51), which we used for enzyme activation in our system.
[f]See supporting material.

indicates a high concentration. In three dimensions, $y_{\max} = 6\ \mu\mathrm{m}$ approximately corresponds to the physiological platelet concentration in human blood, which is about $2.5 \times 10^5\ \mathrm{mm}^{-3}$ (52). We used $x_{\max} = 12\ \mu\mathrm{m}$ and $y_{\max} = 6\ \mu\mathrm{m}$ as default values for the simulations. Detailed calculations are in the supporting material.

When solving the solution-phase equations, periodic boundary conditions are applied at the left ($x = 0$) and right ($x = x_{\max}$) boundaries. A homogeneous Neumann (reflective) boundary condition is imposed at the upper boundary ($y = y_{\max}$). At the lower boundary ($y = 0$), Robin-type boundary conditions are applied on the binding patches, whereas homogeneous Neumann boundary conditions are enforced elsewhere. For the membrane equations, which are solved in one dimension, the left and right boundaries of each binding patch are subject to the homogeneous Neumann boundary condition.

Numerical methods

We employed a uniform Cartesian mesh in both the x- and y-directions over the computational domain $\Omega = [0, x_{\max}] \times [0, y_{\max}]$. Spatial derivatives were approximated using finite differences, with second-order accuracy achieved through central differences for the Laplacian terms. Time discretization for diffusion was performed using the backward Euler method.

To numerically solve the coupled bulk-surface reaction-diffusion system, we adopted an operator-splitting time-stepping scheme that has been used in our previous work (30,31,53). Within each time step, diffusion and reaction processes in the bulk and on the membrane were treated sequentially as follows.

1. Diffusion in the bulk domain was advanced by one time step using the backward Euler method.
2. Reactions in the bulk domain were advanced by one time step using an explicit time-stepping scheme.
3. Diffusion on the membrane was advanced by one time step using the backward Euler method.
4. Reactions on the membrane were advanced by one time step using an explicit time-stepping scheme.

This splitting strategy allows diffusion and reaction processes in the bulk and on the membrane to be treated separately while maintaining numerical stability for the parameter regimes considered in this study. A convergence analysis of the numerical scheme is provided in the supporting material.

Particle-based model

We also adopted a particle-based framework for two key reasons: 1) although the system as a whole contains many molecules, certain species occur at very low concentrations, and 2) the spatial resolution of interest is approximately on the molecular scale. These features make a continuum description less appropriate.

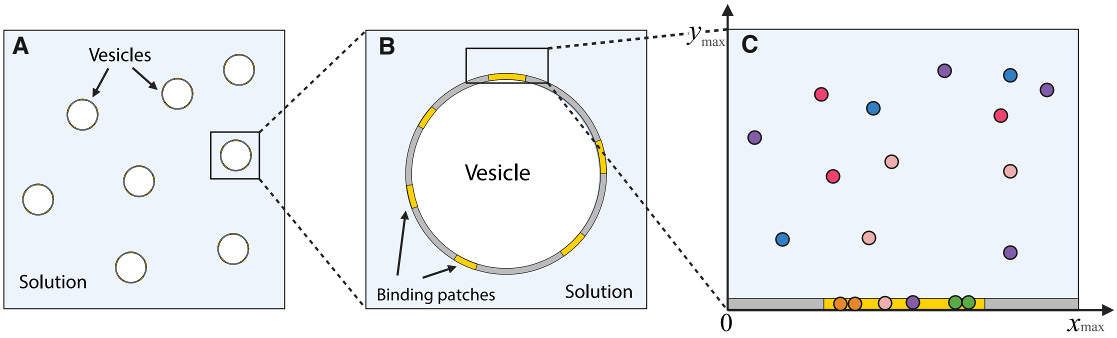

FIGURE 3 Schematic of vesicles with binding patches and the corresponding simulation domain used in the PDE model. (*A*) Schematic of multiple vesicles in the solution domain. (*B*) Enlarged view of one vesicle surface showing binding patches (*yellow*) and non-binding membrane (*gray*). (*C*) Two-dimensional simulation domain used in the PDE model. A portion of the vesicle surface is approximated as a straight-line segment with no curvature, and the adjacent solution region is included to model diffusion and surface exchange.

Particle-based simulations with Smoldyn

Simulations were carried out using Smoldyn (version 2.74), a widely used stochastic reaction-diffusion simulator for biochemical systems (41,54,55). Molecular diffusion was implemented as Gaussian-distributed displacements with zero mean and variance corresponding to the root-mean-square displacement over each time step Δt, reproducing the solution of the diffusion equation for a point-source initial condition. For first-order reactions, the probability of occurrence during Δt was $P = 1 - \exp(-k_1 \Delta t)$, where k_1 is the rate constant. Second-order reactions in three-dimensional were handled by Smoldyn's binding-radius algorithm, in which a binding radius r_b is computed from the diffusion coefficients, the macroscopic association rate constant, and Δt. Two reactants react with probability one when their separation falls within r_b. Reversible reactions were implemented using a two-radius scheme: molecules associate within r_b but, upon dissociation, are placed at an unbinding radius $r_u > r_b$ to avoid immediate reassociation (41). On membrane surfaces, however, the three-dimensional binding-radius algorithm does not apply because the algorithm requires three-dimensional rate constants, whereas two-dimensional analogs are not well defined (56). Although an effective two-dimensional association constant can describe the reaction-limited regime, such values are rarely available and cannot be straightforwardly converted into binding parameters. Therefore, to capture the qualitative dynamics of rapid surface complex formation, a binding radius and collision probability were specified manually. To explicitly track binding-site occupancy, binding patches were represented not as static surface domains but as collections of mobile binding-site particles confined within the patch. This approach makes local depletion explicit and recasts surface binding as a bimolecular particle-particle association. Once bound, enzyme or zymogen particles participate in subsequent 2D bimolecular reactions on the membrane.

Model details and parameters

Smoldyn automatically computed the parameters for first-order and three-dimensional second-order reactions, including solution-solution and solution-membrane associations, based on user-specified Δt, diffusion coefficients, and macroscopic rate constants. Reversible reactions were handled as first-order dissociation events, with products placed at an unbinding radius slightly larger than the forward binding radius. For two-dimensional membrane-membrane associations, binding parameters were manually specified: the binding radius was set to 0.005 μm (the sum of the reactant radii), the binding probability to 0.5 per collision, and the unbinding radius to 0.00501 μm. Dissociation rates were determined from the specified first-order rate constant and Δt. Unless otherwise stated, the default parameters from Table 1 were used. For membrane associations, we applied $r_b = 0.005\ \mu$m, a binding probability of 0.5, and an unbinding radius of 0.00501 μm as previously described, and the simulation time step was set to 0.01 ms. In the particle-based model, all molecules were assumed to be spherical with a radius of 2.5 nm for calculation purposes, consistent with the PDE model assumptions, but they were treated as point-like in simulations without excluded volume effects.

For the particle-based model, we first numerically implemented a quasi-two-dimensional setup to compare qualitative trends with the PDE results under similar geometric constraints. We then performed fully three-dimensional particle-based simulations to demonstrate that the observed qualitative behavior is not solely an artifact of dimensional reduction and to illustrate how the framework can be applied in realistic three-dimensional geometries.

RESULTS

To investigate how the spatial distribution of binding sites on the membrane affects product generation, we considered two distinct scenarios. In the first, all binding sites were grouped into a single patch on the membrane, and we varied the size of the patch, changing the density while keeping the total number of binding sites constant (see Fig. 4 *A*). Pattern 1 represents the least dense distribution, where the binding sites are evenly spread throughout the membrane. In contrast, pattern 4 shows the most compact arrangement, where the binding sites are packed tightly, resulting in the highest possible binding-site density. In the second scenario, we fix the density of the binding patches but distribute the binding sites across multiple subpatches of equal size, which are evenly spread across the membrane surface (see Fig. 4 *B*). To systematically vary spatial organization, the total binding area is partitioned into an increasing number of equally sized, maximally dense sub-patches that are evenly distributed across the membrane.

Across all scenarios, binding patches were assumed to be fixed in size, density, and spatial location over the timescale of the simulations. To facilitate consistent comparison across different spatial configurations, enzyme generation efficiency was evaluated using the spatially averaged solution-phase enzyme concentration. Specifically, reaction efficiency was characterized using two metrics: the peak enzyme concentration and the time required to reach this peak.

Spatiotemporal dynamics of solution-phase Z_1 depletion and E_1 generation

Fig. 5 *A* shows representative simulation results as spatial concentration profiles of solution-phase Z_1 and E_1 at selected time points. In this example, a single binding patch is located at the center of the bottom boundary. Initially, Z_1 is abundant,

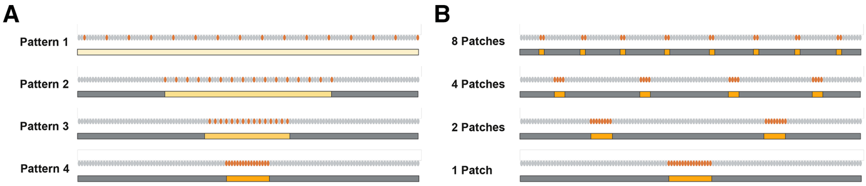

FIGURE 4 Illustration of different binding-site distributions on the membrane surface. Each distribution is shown using two representations: the top row presents schematic diagrams of the membrane, where yellow particles represent binding sites, and gray particles indicate nonbinding membrane areas; the bottom row shows binding-site density maps, where yellow indicates regions containing binding patches, and brightness reflects local density. (*A*) A single binding patch with varying size and density, while keeping the total number of binding sites constant. From pattern 1 to pattern 4, the patch spans 1, 1/2, 1/4, and 1/8 of the membrane length, respectively. Correspondingly, the binding-site densities are $b_{max}/8$, $b_{max}/4$, $b_{max}/2$, and b_{max}, where $b_{max} = 10\ \text{pmol/cm}^2$ denotes the theoretical upper bound of binding-site density. (*B*) Multiple binding patches with fixed density and varying numbers of patches, while maintaining the same total number of binding sites. The density of all binding patches is fixed at the upper bound $b_{max} = 10\ \text{pmol/cm}^2$. The total length of all binding patches combined equals 1/8 of the membrane length in each distribution.

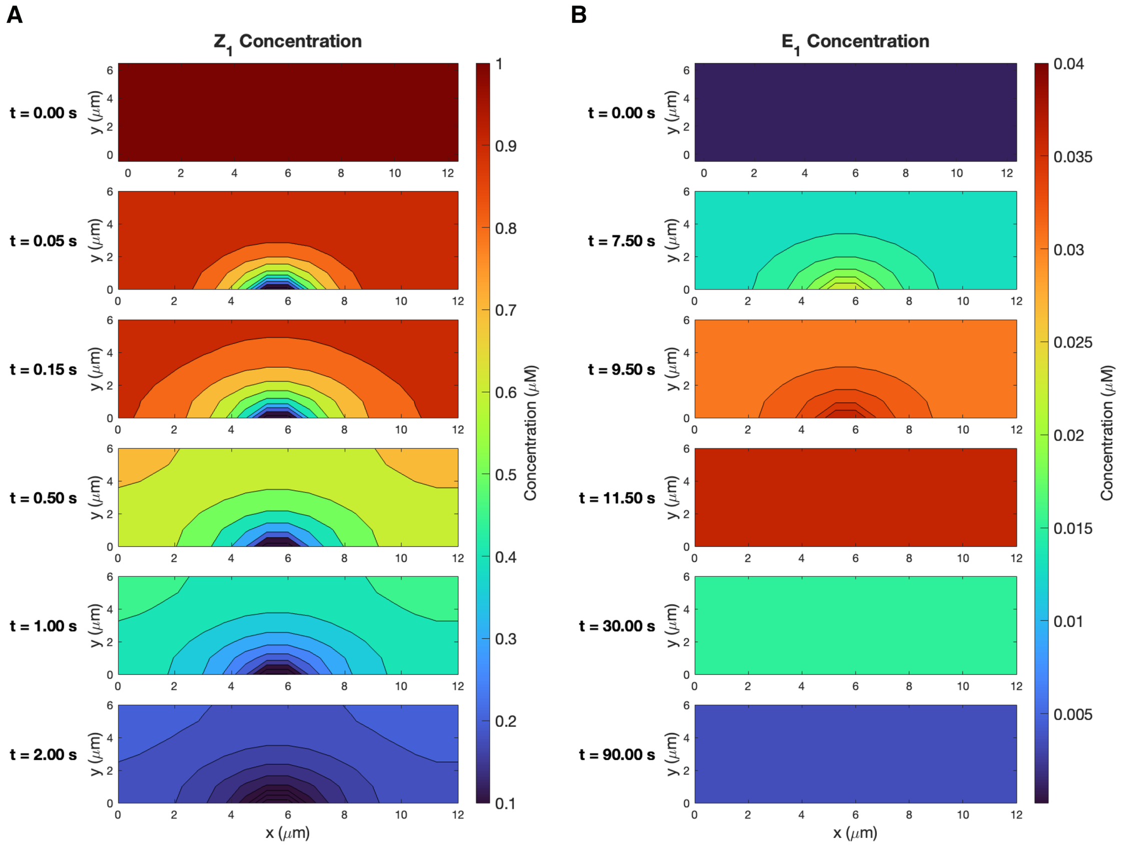

FIGURE 5 Spatial concentration profiles of Z_1 and E_1 as a function of time, for a single binding patch located at $y = 0$ μm and $x \in [5.25, 6.75]$ μm. (*A*) Z_1 profiles at t = 0.00, 0.05, 0.15, 0.50, 1.00, and 2.00 s (*B*) E_1 profiles at time t = 0.00, 7.50, 9.50, 11.50, 30.00, and 90.00 s.

but shortly thereafter, the binding patch acts as a sink that adsorbs Z_1 from solution. By $t = 2$ s, solution-phase Z_1 is largely depleted. In Fig. 5 *B*, no E_1 is present at $t = 0$ s; however, by $t = 7.5$ s, E_1 has begun to be released from the binding patch, which now functions as a source. The solution-phase E_1 concentration reaches a maximum at approximately $t = 11.5$ s, after which it decreases due to inhibition. These results highlight a clear difference in the timescales of Z_1 depletion and E_1 generation.

Effect of binding-site distribution on enzyme generation with a single binding patch

We first investigated the effect of binding-site distribution by considering a single binding patch whose size and density were varied while keeping the total number of binding sites constant. These configurations correspond to the cases depicted in Fig. 4 *A*.

Enzyme generation increases as binding patch size and sparsity increase in the transport-limited regime

Increasing the membrane association rate constant between enzymes and zymogens to 3 $\text{cm}^2/(\text{pmole}\cdot\text{s})$ shifts the system into a transport-limited regime, as reactant association on the membrane becomes sufficiently fast that transport in the solution becomes the rate-limiting step. Under these conditions, we observed a monotonic relationship between enzyme generation efficiency and binding patch compactness, with peak enzyme concentration decreasing and time to peak increasing as patch size decreases and density increases (Fig. 6). The patch with the largest area and lowest density (pattern 1 in Fig. 4 *A*) yielded the highest efficiency, characterized by both a higher peak enzyme concentration and a shorter time to peak (Fig. 6). In contrast, progressively smaller and denser patches resulted in reduced enzyme generation efficiency. This behavior arises because enzyme activation occurs exclusively on the membrane surface. Larger binding patches reduce the average search time for diffusing solution-phase molecules to encounter reactive sites, whereas smaller, denser patches impose a greater transport penalty in the bulk solution in the transport-limited regime.

Enzyme generation increases as binding patch size and sparsity decrease in the reaction-limited regime

By increasing the solution diffusivity to $D_{sol} = 1 \times 10^{-4}$ cm^2/s, we shifted the system into a regime in which transport in the solution was no longer rate limiting, whereas membrane reactions became the dominant limiting process. In this regime, enzyme activity on the membrane governed the overall reaction efficiency. The largest and least dense

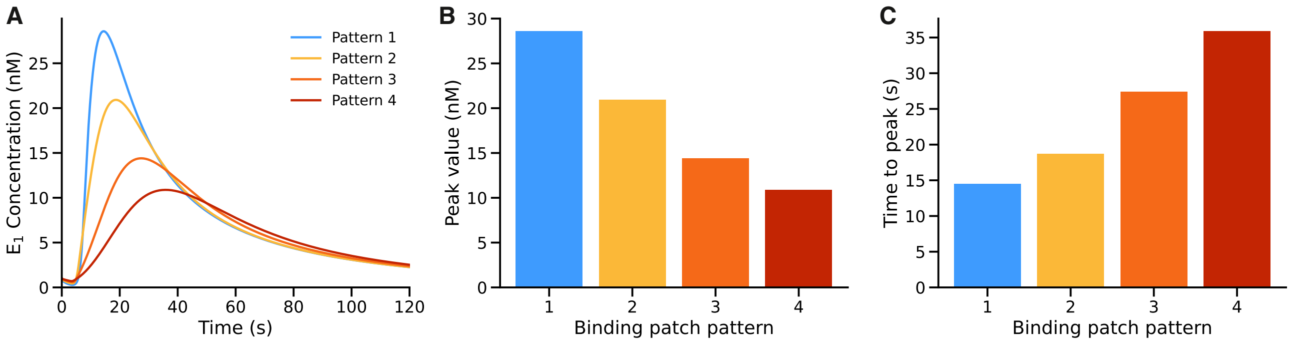

FIGURE 6 PDE simulation results in the transport-limited regime: enzyme generation with single binding patch of varying density and size, while keeping the total amount of binding sites held constant. (*A*) Time courses of enzyme concentration for different binding-site distributions with fixed total binding sites but varying patch size and density. (*B*) Bar plot showing the peak enzyme concentration for each binding-site distribution. (*C*) Bar plot showing the time to reach peak enzyme concentration for each distribution.

binding patch (pattern 1 in Fig. 4 *A*) exhibited the lowest efficiency, with both the smallest peak enzyme concentration and the longest time to peak. As the binding-site density increased and patch size decreased, enzyme generation efficiency improved progressively (Fig. 7). Notably, the smallest and densest binding patch (pattern 4 in Fig. 4 *A*) achieved the highest efficiency despite its limited area.

In the reaction-limited regime, molecules in the solution can reach the surface binding sites in comparable time, regardless of patch size. However, once bound, the local surface densities differ across patterns. According to the law of mass action, higher densities of both enzyme and zymogen accelerate their interactions, thereby enhancing reaction efficiency.

Transport-reaction trade-offs give rise to an optimal binding patch size in the transport-influenced regime

For the default value of the solution-phase diffusivity and the association rates between enzymes and zymogens on the membrane, the PDE simulations show a nonmonotonic relationship between both peak and time to peak and patch size (Fig. 8). This behavior highlights the balance between the transport of solution-phase molecules to the binding sites and the enzymatic reactions on the membrane once molecules become surface bound. Among the tested patterns, binding patch pattern 2 (intermediate patch size) led to the highest peak value with a relatively short time to peak. Its size allowed solution-phase molecules to reach the surface quickly while maintaining a sufficiently high binding-site density.

Effect of binding-site distribution on enzyme generation with varied number of patches

From the previous simulation results, we observed that efficient product generation is favored by two conditions: binding sites packed densely within each patch and patches distributed broadly across the membrane. The first condition enables a fast reaction rate according to mass-action kinetics, whereas the second facilitates ready access of solution proteins to the binding sites. Motivated by these findings, we next investigate the binding-site distribution shown in Fig. 4 *B*.

PDE model predicts monotonically increasing enzyme generation with higher number of smaller patches

Here, we fixed the total number of binding sites and subdivided them into smaller patches, as illustrated in Fig. 4 *B*. The binding site density is set to its maximum value.

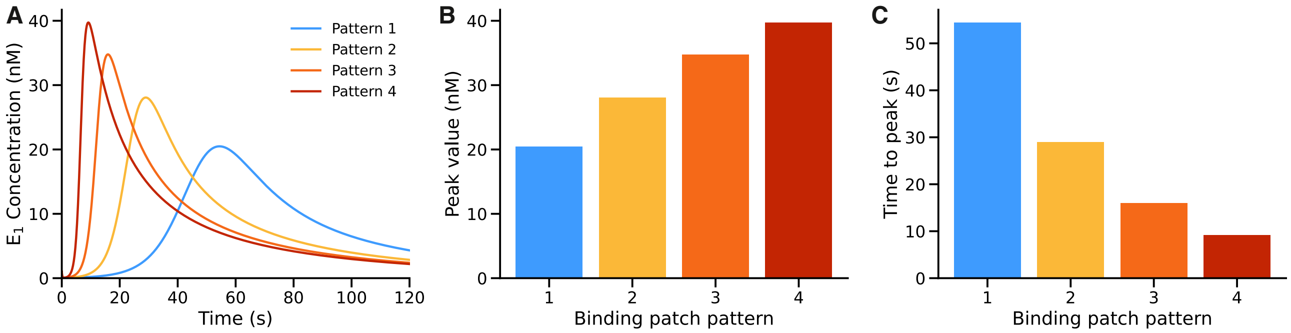

FIGURE 7 PDE simulation results in the reaction-limited regime: enzyme generation with single binding patch of varying density and size, while keeping the total amount of binding sites held constant. (*A*) Enzyme generation curves for varying binding-site distribution with fixed density and varying patch size. (*B*) Bar plot of peak enzyme concentration for different binding-site distributions. (*C*) Bar plot of time to reach peak enzyme concentration for different binding-site distributions.

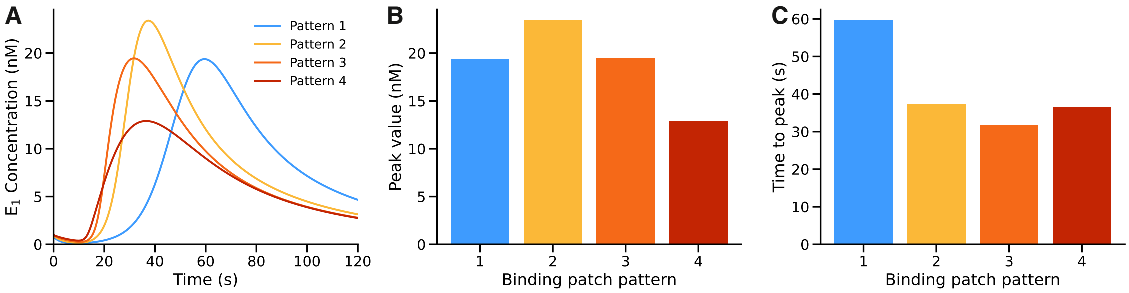

FIGURE 8 PDE simulation results in the transport-influenced regime: enzyme generation with single binding patch of varying density and size, while keeping the total amount of binding sites held constant. (*A*) Enzyme generation curves for varying binding-site distribution with fixed density and varying patch size. (*B*) Bar plot of peak enzyme concentration for different binding-site distributions. (*C*) Bar plot of time to reach peak enzyme concentration for different binding-site distributions.

As shown in Fig. 9, increasing the number of patches produces enzyme generation curves with higher peaks and shorter times to peak. Both quantities increase (or decrease, respectively) monotonically with patch number and eventually approach limiting values. Similar trends are observed for the physiological diffusion coefficient (not shown).

This monotonic behavior can be explained by transport limitations in the solution. Because the binding-site density is fixed, transport of solution-phase species to the membrane remains the rate-limiting step. With many small patches distributed across the surface, solution molecules encounter binding sites more readily, accelerating enzyme generation.

It is notable that at very high patch numbers, the PDE model still predicts efficient enzyme generation, because each patch is represented as a finite area with nonzero binding-site density under the continuum assumption. In reality, subdividing the total binding sites into many patches would leave only one, or even zero, binding sites per patch, rendering surface-bound proteins effectively isolated. Without cross-patch interactions, reactions would be strongly suppressed. This discrepancy between continuum predictions and the expected molecular-scale outcome illustrates the limitation of PDE models once patch sizes approach molecular dimensions. To address this limitation, we employed quasi-two-dimensional particle-based simulations that explicitly represent individual molecules and their interactions.

Quasi-two-dimensional particle-based simulations show enzyme generation is nonmonotonic with patch number

For qualitative comparison with the PDE simulations, we used a quasi-two-dimensional particle-based domain matching the PDE dimensions in the x and y directions and extending 0.02 μm in the z direction. The thin depth ensured adequate enzyme numbers at the specified concentration while preserving an effectively two-dimensional geometry.

Given the system volume and prescribed concentrations, the initial particle counts were 1 E_1, 1 E_2, 1000 Z_1, and 1000 Z_2. For each condition, enzyme generation curves represent averages over 50 independent stochastic simulations. Particle numbers were converted to concentrations by dividing by the system volume. Consistent with the PDE simulations, the binding patches occupied one-eighth of the membrane surface, corresponding to a total of 1800 binding sites (Fig. 4 *B*).

The quasi-two-dimensional particle-based simulations revealed a nonmonotonic relationship between enzyme generation efficiency and patch number under fixed total binding sites (Fig. 10). The most efficient enzyme generation was achieved between 40 and 200 patches. For small number

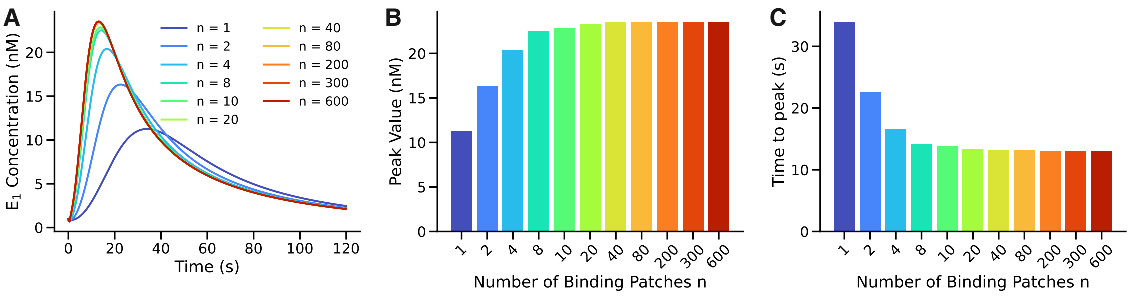

FIGURE 9 PDE model results: enzyme generation with multiple patches of fixed density and varying number of patches, while keeping the total amount of binding sites held constant. (*A*) Enzyme generation curves for different binding-site distributions with fixed density and varying patch size. (*B*) Bar plot of peak enzyme concentration for different binding-site distributions. (*C*) Bar plot of time to reach peak enzyme concentration for different binding-site distributions.

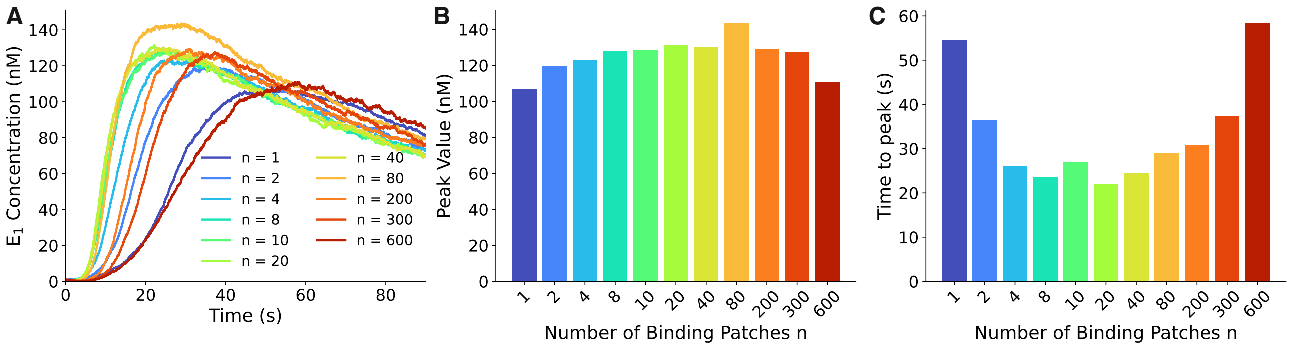

FIGURE 10 Quasi-two-dimensional particle-based model results: enzyme generation with binding patches of varying density and patch sizes. (*A*) Enzyme generation curves for varying binding-site distribution with fixed density and varied patch size. (*B*) Bar plot of peak product concentration for different binding-site distributions. (*C*) Bar plot of time to reach peak product concentration for different binding-site distributions.

of patches, enzyme generation is transport limited; an intermediate number of patches improves surface capture and accelerates enzyme generation; for the high number of patches, further subdivision left too few binding sites per patch, which reduced local reactant availability and rendered the system reactant-encounter-limited.

Three-dimensional particle-based simulations confirm nonmonotonic relationship between enzyme generation and number of patches

To verify that the nonmonotonic dependence observed in quasi-two-dimensional simulations is not an artifact of the reduced dimensionality, we next performed fully three-dimensional particle-based simulations.

The three-dimensional simulations used a cubic solution domain that fully surrounds a single vesicle and resolves all three spatial dimensions. To simplify the geometry and enable more precise control over the placement and size of binding patches, the vesicle (radius 75 nm) was approximated as a cube (Fig. 11); preserving total surface area yields a side length of $\sim$0.108 μm. The vesicle was positioned at the center of a larger cubic solution box, and the solution occupied the region between the vesicle surface and the outer box. The vesicle surface was reflective except at designated binding patches, which captured enzymes and zymogens, whereas periodic boundary conditions were imposed at the outer box faces. We verified that the cubic-vesicle approximation had negligible effect on the results (see supporting material).

Binding patches were modeled as circular regions containing a fixed number of diffusing binding-site particles, with patch radii determined by the number of sites per patch. Assuming that 10% of membrane lipids are PS and they all contribute as binding sites, we estimated $\sim$360 binding sites per vesicle. Details of the calculations for vesicle side length, solution domain size, and patch radii are provided in the supporting material.

To more accurately capture inhibition dynamics, we explicitly modeled the inhibitor species (In) and the corresponding inhibited enzyme complexes: E_1 : In and E_2 : In in solution, and E_1^{m} : In and E_2^{m} : In on the membrane. These complexes were assumed to compete with active enzymes and zymogens for available binding sites. The new reactions describing this competition were as follows:

$$E_1 : In + B^{avail} \underset{k_{1off}}{\overset{k_{1on}}{\rightleftharpoons}} E_1^{\mathrm{m}} : In, \qquad E_2 : In + B^{avail} \underset{k_{2off}}{\overset{k_{2on}}{\rightleftharpoons}} E_2^{\mathrm{m}} : In.$$

Now that inhibitors are explicitly modeled, we specified the second-order inhibition rate constant between enzymes and inhibitors to be $0.01/(\mathrm{nM} \cdot \mathrm{s})$. The dissociation rate from the membrane is changed to 1/s. The initial particle numbers were: 4 E_1, 4 E_2, 400 Z_1, 400 Z_2, 1000 In, and 360 B (binding sites), with all other species starting at zero. Across simulations, two outcomes were observed: in some cases, enzyme generation was initiated, whereas in others, inhibition dominated, and enzyme generation failed to even begin. To capture stochastic variability, we performed 200 independent simulations with different random

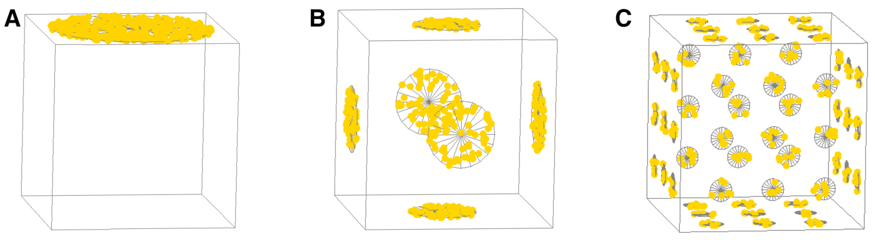

FIGURE 11 Snapshots of simulated cubic vesicles with different binding patch distribution, while maintaining a constant total number of binding sites. Yellow particles denote binding sites confined within circular patches on the vesicle surface. (*A*) One single large patch. (*B*) Six medium-sized patches, one on each face. (*C*) Fifty-four small patches, with nine per face.

seeds for each patch distribution. We retained ∼100 runs in which enzyme generation was initiated and used these for subsequent analysis.

The fully three-dimensional particle-based simulations reproduced the nonmonotonic dependence of enzyme generation efficiency on patch number observed in the quasi-two-dimensional setup (Fig. 12). Efficiency increased as the number of patches grew but declined once the number exceeded around 24. This agreement confirms that the turnover behavior is not an artifact of dimensional reduction but rather reflects the discrete distribution of binding sites on the vesicle surface.

Summary of findings

In this study, we simulated a system of surface-dependent reactions with a positive feedback loop, focusing on how the spatial distribution of binding sites on a lipid vesicle membrane influences reaction dynamics. We employed both a continuum PDE model and a particle-based stochastic model to investigate these effects across different spatial resolutions.

Our main findings are as follows: 1) given a fixed total number of binding sites grouped into a single patch, placing them densely in a small region leads to the fastest product generation when the catalytic activities on the membrane is the limiting step (fast transport in the solution); 2) in contrast, when transport in the surrounding solution is the limiting factor, a large, sparse patch is more efficient for product generation; and 3) in a nonlimiting regime, where neither reaction on the membrane nor transport dominates, the product generation efficiency represents a balance between the two processes. Furthermore, when the total number of binding sites is fixed but distributed into multiple binding patches, 4) the PDE model predicts increasing efficiency as the number of patches increases. However, this last prediction becomes inaccurate when the number of binding sites per patch becomes too low, due to the limitations of representing binding sites and proteins as continuous fields.

The particle-based model accurately captured the discrete nature of the system. It revealed a nonmonotonic trend: efficiency initially increases with the number of binding patches but eventually decreases when the patches become too small. The initial increase arises because distributing more patches across the surface allows molecules in the solution to encounter binding sites more readily. However, as the number of patches continues to grow, each patch contains fewer binding sites, leading to fewer reactant molecules being recruited and retained near each surface-bound protein, which reduces the overall reaction efficiency.

DISCUSSION

In this study, we investigated how the spatial distribution of membrane binding sites influences the dynamics of a positive-feedback surface reaction system. By using both continuum PDE and particle-based models, we identified distinct regimes in which reaction efficiency is limited either by catalytic turnover on the membrane or by transport in the surrounding solution. Furthermore, we found that the number and spatial organization of binding patches are critical: particle-based simulations revealed an optimal distribution that maximizes product generation, a feature not captured by continuum models.

Continuum model versus discrete model

Although continuum models such as PDE and ODE frameworks are powerful and computationally efficient for simulating chemical and biological systems, there are several important scenarios where stochastic discrete models become necessary. These include 1) systems with low concentrations of molecules, where stochastic fluctuations significantly influence system behavior (57); 2) situations where spatial resolution approaches molecular dimensions, rendering the continuum approximation invalid; and 3) systems in which stochasticity drives qualitatively distinct dynamics, such as noise-induced switching or pattern formation (58).

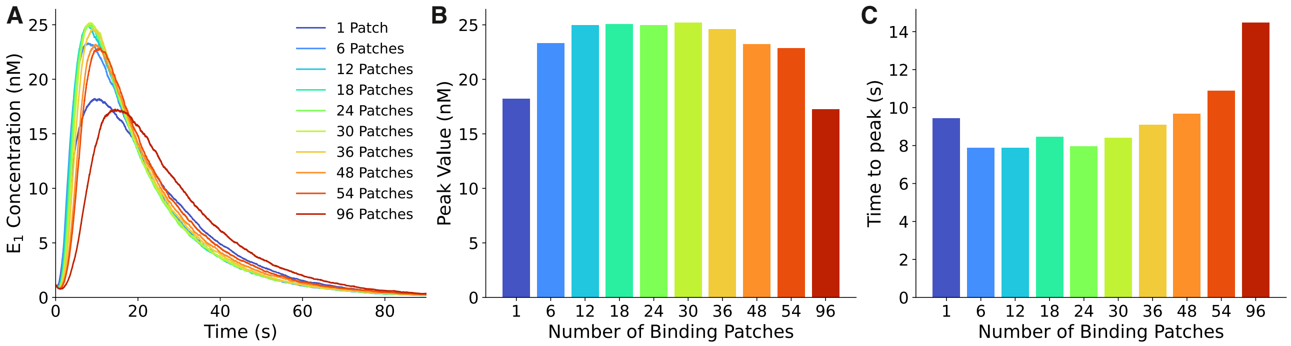

FIGURE 12 Particle-based model results: enzyme generation with binding patches of varying density and patch sizes. (*A*) Enzyme generation curves for varying binding-site distributions with fixed density and varied patch size. (*B*) Bar plot of peak product concentration for different binding-site distributions. (*C*) Bar plot of time to reach peak product concentration for different binding-site distributions.

In this study, we employed both types of models to investigate surface-dependent reactions across spatial scales. The PDE model was applied at the micron scale, representative of a human platelet, and remains accurate when binding patches are on the order of microns. However, when the patch size approached nanometer dimensions, i.e., comparable to individual molecules, the continuum model became inaccurate due to its inability to capture molecular discreteness. To address this limitation, we used a particle-based model to simulate reactions at the nanoscale. This approach explicitly accounts for the discrete nature of molecules and binding events, providing a more accurate description when patch sizes are small. Our simulations suggest that an optimal binding-site distribution may correspond to approximately 24 patches distributed over the surface, with about 15 binding sites per patch. The precise optimum, however, is likely to vary depending on the biological system being modeled and parameter values used.

Particle-based analog to the template effect

Our particle-based simulations revealed a nonmonotonic dependence of reaction efficiency on the distribution of surface binding sites. When binding sites were concentrated within a limited patch, substrate molecules frequently encountered the enzyme, leading to enhanced reaction rates. However, when binding sites were distributed more sparsely across many small or isolated patches, substrates were absorbed by regions lacking enzyme and became spatially separated from catalytic sites, which reduced the effective reaction rate. This bell-shaped dependence mirrors the classical template effect first described in coagulation studies, where excess lipid or heparin acts as a nonproductive sink for substrate, thereby diluting enzyme-substrate encounters (51). More recently, Madrigal et al. (29) demonstrated with a two-compartment mathematical model that enzyme-deficient vesicles added to enzyme-positive vesicles sequestering zymogen and thereby reproduced the template effect in lipid-mediated coagulation. Our particle-based model provides a mechanistic analog at the microscopic scale, explicitly showing how spatial sequestration of substrate by nonproductive sites attenuates overall enzymatic activity.

Spatial distribution of phosphatidylserine on platelet membranes

In resting platelets, the outer leaflet contains little or no PS. Under strong stimulation, only a subpopulation of about 5%–10% of platelets externalizes PS, with the outer leaflet reaching 8%–15% PS of total outer-leaflet phospholipids (59–61). PS distribution on activated platelet surfaces is not fully random but arranged in specific patterns. Experimental studies have reported that many procoagulant platelets exhibit cap-like regions with dense PS enrichment; in some cases, multiple PS-rich caps are observed (62,63). Moreover, it has been hypothesized that flow conditions may contribute to the formation of such cap-like structures (64). Beyond platelets, PS exposure is a more general feature of procoagulant membrane remodeling. For example, PS-rich microparticles shed from red blood cells and platelets in sickle cell disease provide abundant PS-rich surfaces for tenase and prothrombinase assembly, and they are thought to contribute to the thrombotic risk associated with vaso-occlusion (65,66). Similarly, tumor cells and tumor-derived vesicles frequently externalize PS, which has been linked to a prothrombotic state in cancer patients and the development of cancer-associated thrombosis (67,68). These broader contexts highlight that PS exposure on cellular membranes and vesicles is not unique to platelet activation, but it represents a generalizable feature of pathophysiological coagulation. Although platelets exhibit nonrandom, patch-like PS distribution, other cell types such as red blood cells and tumor cells contribute through the shedding of PS-rich vesicles. Future studies could therefore benefit from integrating insights across these systems to delineate how PS topography and distribution regulate thrombin generation in both normal hemostasis and disease.

Our simulation results suggest that having a single large patch with a high percentage of PS is not the optimal arrangement for maximizing reaction efficiency on a vesicle surface. However, in physiological platelets, additional physical constraints, such as cytoskeletal interactions, membrane organization, and overall morphology, limit how negatively charged lipids can be distributed, preventing them from adopting the theoretically optimal configuration we propose. Our simplified model does not take these complex structural features into account. Nevertheless, this view is consistent with studies in nonplatelet systems: in synthetic liposomes, PS displays a nonuniform, clustered organization on the outer membrane surface, a feature revealed by fluorescence microscopy and lipid labeling that markedly influences protein binding (69). Similarly, in apoptotic cells, externalized PS is spatially distinct from lipid raft domains (70), suggesting that raft microdomains may further compartmentalize the sites of PS exposure. Platelets themselves contain abundant cholesterol- and sphingolipid-rich lipid rafts that serve as signaling platforms for receptors such as GPVI and GPIb-IX-V (71,72), and the presence of these raft domains may spatially restrict where PS can be externalized.

In the current work, we made an idealized assumption that PS-rich binding patches are immobile over the timescale of the reactions considered. This simplification allows us to isolate the effect of spatial distribution on reaction efficiency without introducing additional complexity from membrane remodeling. Incorporating dynamic lipid redistribution and its coupling to protein binding is an important extension of the present framework and will be explored in future work.

Application to platelet-inspired synthetic hemostats

Our modeling and simulation approaches can also provide design guidance to the optimization of platelet-inspired synthetic lipid based hemostatic particles, such as the platelet-mimicking procoagulant nanoparticles (PPNs) recently reported by Sekhon et al. (11). Development and translation of such synthetic platelet systems are of great clinical interest as they can provide a donor-independent platelet surrogate option for transfusion management of bleeding complications, especially where donor-derived platelet products are of limited availability (73). To this end, the procoagulant lipid particles incorporated distearoyl phosphatidylserine (DSPS) as the PS-presenting lipid component in the membrane shell of the particles. In experimental characterization of these particles regarding their capability of thrombin generation, an interesting observation was that the compositional amount (mol %) of DSPS utilized in the particle manufacture had a nonmonotonic dependence on PS content: peak thrombin generation and the shortest time to peak were observed when 10%–15 mol % of DSPS was incorporated into the PPN membrane (Fig. S6 in (11)). To better understand the underlying mechanisms, we performed particle-based simulations with varying percentages of PS (results not shown). Specifically, we modulated the number of binding-site particles and the corresponding size of PS-rich patches. A nonmonotonic dependence on PS content was observed in the peak level of product generation, whereas the time to peak remained monotonic. This behavior contrasts with the results reported in the Sekhon et al. paper, where both peak thrombin generation and time to peak exhibited nonmonotonic trends. The discrepancy likely arises from a key mechanistic difference: in coagulation, thrombin does not bind to the PS surface but is released into solution upon its generation by the prothrombinase complex. In our simulation, the enzyme remains capable of binding to PS-rich surfaces. As the PS content increases, more enzyme molecules become bound, and due to their binding affinity, they tend to remain associated with the surface even after catalysis. Unlike thrombin, which diffuses away after formation, the enzyme in our model does not readily dissociate from the surface, resulting in a PS-dependent surface retention effect that alters catalytic dynamics. To more accurately capture these dynamics, future simulations incorporating a reaction system more representative of the coagulation network will be necessary. Despite this mechanistic difference in the reaction system, we showed that when holding the total number of binding sites constant, varying the spatial distribution of PS-rich regions (binding patches) modulates reaction efficiency. Our simulations revealed nonmonotonic behavior: for instance, in the multipatch setting, increasing the number of patches initially improved catalytic efficiency, but excessive fragmentation led to performance decline, implying the existence of an optimal PS distribution. In their experimental work, a similar nonmonotonic dependence was observed for both time to peak and peak thrombin generation as a function of PS percentage, with an optimal range around 10%–15% yielding the fastest product generation and shortest time to peak. Taken together, these insights underscore the importance of both the total amount as well as the spatial organization of PS in designing effective synthetic platelet-like nanoparticles. To this end, our ongoing and future work will focus on correlating PS concentration, patch area, and patch density to optimize co-assembly with coagulation factor molecules so as to maximize thrombin generation. Such analyses will guide the refinement of procoagulant synthetic platelet designs such that minimal dosing of such systems can be utilized to achieve maximized therapeutic effect.

Parameterizing two-dimensional surface reactions in particle-based models

Accurately parameterizing surface-bound bimolecular reactions in two dimensions presents a well-known challenge. Unlike in three dimensions, where association rate constants have clear physical interpretations, two-dimensional reaction rates lack such well-defined formulations. Although empirical approaches exist, e.g., estimating a macroscopic rate constant from a particle-based simulation using fixed binding radii and reaction probabilities (58), these are typically employed to reconcile stochastic simulations with continuum models and to explore the role of stochasticity.

However, achieving precise agreement between the particle-based model and the PDE model was not our objective. Rather than estimating macroscopic kinetics from fixed microscopic parameters, we aimed to use known macroscopic rates as reference points for selecting suitable microscopic parameters (i.e., binding probability, unbinding radius). Systematically deriving microscopic values from macroscopic rate constants in two dimensions remains challenging, particularly in systems where the reported apparent rates are three-dimensional, even when describing interactions between membrane-bound species.

To avoid this parameterization challenge in the present study, we did not attempt to rigorously derive microscopic parameters from macroscopic rate constants or to achieve quantitative agreement between the PDE and particle-based models. Instead, particle-based simulations were used to assess whether the qualitative trends predicted by the continuum model persist when molecular discreteness and stochasticity are explicitly represented. Developing systematic parameterization strategies that link microscopic reaction rules in particle-based simulations to effective macroscopic kinetics in two dimensions remains an important open problem. Such approaches would be required to enable quantitative matching between continuum and discrete models and to facilitate direct comparison across modeling frameworks. Addressing this challenge is beyond the scope of the present

work and represents a natural direction for future studies aimed at integrating PDE-based and particle-based descriptions of surface-mediated biochemical reactions.

DATA AND CODE AVAILABILITY

All codes are available at https://github.com/LeidermanLab/ContinuumDiscreteLipidVesicleModeling.

ACKNOWLEDGMENTS

This work was, in part, supported by the National Institutes of Health (R01 HL151984, K.L., 2R01 HL121212, A.S.G) and the National Science Foundation CAREER (DMS-1848221, K.L.).

AUTHOR CONTRIBUTIONS

H.C. carried out all simulations. H.C., A.S.G., and K.L. designed the research, analyzed the data, and wrote the article.

DECLARATION OF INTERESTS

The authors declare no competing interests.

SUPPORTING MATERIAL

Supporting material can be found online at https://doi.org/10.1016/j.bpj.2026.03.015.

REFERENCES

1. Hoffman, M., and D. M. Monroe. 2001. A Cell-Based Model of Hemostasis. *Thromb. Haemost.* 85:958–965.
2. Reddy, E. C., and M. L. Rand. 2020. Procoagulant Phosphatidylserine-Exposing Platelets in Vitro and in Vivo. *Front. Cardiovasc. Med.* 7:15.
3. Mackman, N., and M. Taubman. 2009. Tissue Factor. *Arterioscler. Thromb. Vasc. Biol.* 29:1986–1988.
4. Dho, S. H., J. C. Lim, and L. K. Kim. 2018. Beyond the Role of CD55 as a Complement Component. *Immune Netw.* 18:e11.
5. Charles, A., J. Janeway, …, M. J. Shlomchik. 2001. Immunological Memory. *In* Immunobiology: The Immune System in Health and Disease, 5th Edition Garland Science.
6. Rollins, S. A., and P. J. Sims. 1990. The Complement-Inhibitory Activity of CD59 Resides in Its Capacity to Block Incorporation of C9 into Membrane C5b-9. *J. Immunol.* 144:3478–3483.
7. Dunkelberger, J. R., and W.-C. Song. 2010. Complement and Its Role in Innate and Adaptive Immune Responses. *Cell Res.* 20:34–50.
8. Lanzavecchia, A., G. Iezzi, and A. Viola. 1999. From TCR Engagement to T Cell Activation: A Kinetic View of T Cell Behavior. *Cell.* 96:1–4.
9. Sewell, A. K. 2012. Why Must T Cells Be Cross-Reactive? *Nat. Rev. Immunol.* 12:669–677.
10. Lynch, T. J., D. W. Bell, …, D. A. Haber. 2004. Activating Mutations in the Epidermal Growth Factor Receptor Underlying Responsiveness of Non–Small-Cell Lung Cancer to Gefitinib. *N. Engl. J. Med.* 350:2129–2139.
11. Sekhon, U. D. S., K. Swingle, …, A. Sen Gupta. 2022. Platelet-Mimicking Procoagulant Nanoparticles Augment Hemostasis in Animal Models of Bleeding. *Sci. Transl. Med.* 14:eabb8975.
12. Davie, E. W., K. Fujikawa, and W. Kisiel. 1991. The Coagulation Cascade: Initiation, Maintenance, and Regulation. *Biochemistry.* 30:10363–10370.
13. Weisel, J. W. 2007. Structure of Fibrin: Impact on Clot Stability. *J. Thromb. Haemost.* 5:116–124.
14. Ruggeri, Z. M. 2003. Von Willebrand Factor, Platelets and Endothelial Cell Interactions. *J. Thromb. Haemost.* 1:1335–1342.
15. Mackman, N. 2004. Role of Tissue Factor in Hemostasis, Thrombosis, and Vascular Development. *Arterioscler. Thromb. Vasc. Biol.* 24:1015–1022.
16. Heemskerk, J. W. M., N. J. A. Mattheij, and J. M. E. M. Cosemans. 2013. Platelet-based Coagulation: Different Populations, Different Functions. *J. Thromb. Haemost.* 11:2–16.
17. Monroe, D. M., and M. Hoffman. 2006. What Does It Take to Make the Perfect Clot? *Arterioscler. Thromb. Vasc. Biol.* 26:41–48.
18. Swieringa, F., H. M. H. Spronk, …, P. E. J. van der Meijden. 2018. Integrating Platelet and Coagulation Activation in Fibrin Clot Formation. *Res. Pract. Thromb. Haemost.* 2:450–460.
19. Hoffman, M., and D. M. Monroe. 2007. Coagulation 2006: A Modern View of Hemostasis. *Hematol. Oncol. Clin. North Am.* 21:1–11.
20. Zwaal, R. F., and A. J. Schroit. 1997. Pathophysiologic Implications of Membrane Phospholipid Asymmetry in Blood Cells. *Blood.* 89:1121–1132.
21. Hemker, H. C., J. L. van Rijn, …, R. F. Zwaal. 1983. Platelet Membrane Involvement in Blood Coagulation. *Blood Cells (N. Y.).* 9:303–317.
22. Kempton, C. L., M. Hoffman, …, D. M. Monroe. 2005. Platelet Heterogeneity: Variation in Coagulation Complexes on Platelet Subpopulations. *Arterioscler. Thromb. Vasc. Biol.* 25:861–866.
23. Paul, D., and J. H. Morrissey. 2022. Stoichiometric Analysis Reveals a Unique Phosphatidylserine Binding Site in Coagulation Factor X. *J. Thromb. Haemost.* 20:600–604.
24. Morrissey, J. H., V. Pureza, …, E. Tajkhorshid. 2008. Blood Clotting Reactions on Nanoscale Phospholipid Bilayers. *Thromb. Res.* 122:S23–S26.
25. Fogelson, A. L., and A. L. Kuharsky. 1998. Membrane Binding-site Density Can Modulate Activation Thresholds in Enzyme Systems. *J. Theor. Biol.* 193:1–18.
26. Beltrami, E., and J. Jesty. 2001. The Role of Membrane Patch Size and Flow in Regulating a Proteolytic Feedback Threshold on a Membrane: Possible Application in Blood Coagulation. *Math. Biosci.* 172:1–13.
27. Kuharsky, A. L., and A. L. Fogelson. 2001. Surface-Mediated Control of Blood Coagulation: The Role of Binding Site Densities and Platelet Deposition. *Biophys. J.* 80:1050–1074.
28. Bungay, S. D., P. A. Gentry, and R. D. Gentry. 2003. A Mathematical Model of Lipid-Mediated Thrombin Generation. *Math. Med. Biol.* 20:105–129.
29. Madrigal, J., D. M. Monroe, …, K. Leiderman. 2024. Modeling the Distribution of Enzymes on Lipid Vesicles: A Novel Framework for Surface-Mediated Reactions in Coagulation. *Math. Biosci.* 374:109229.
30. Leiderman, K., and A. L. Fogelson. 2011. Grow with the Flow: A Spatial–Temporal Model of Platelet Deposition and Blood Coagulation under Flow. *Math. Med. Biol.* 28:47–84.
31. Leiderman, K., and A. L. Fogelson. 2013. The Influence of Hindered Transport on the Development of Platelet Thrombi Under Flow. *Bull. Math. Biol.* 75:1255–1283.
32. Berg, H. C., and E. M. Purcell. 1977. Physics of Chemoreception. *Biophys. J.* 20:193–219.
33. Handy, G., and S. D. Lawley. 2021. Revising Berg-Purcell for Finite Receptor Kinetics. *Biophys. J.* 120:2237–2248.
34. Lawley, S. D., A. E. Lindsay, and C. E. Miles. 2020. Receptor Organization Determines the Limits of Single-Cell Source Location Detection. *Phys. Rev. Lett.* 125:018102.

35. Lawley, S. D., and J. B. Madrid. 2020. A Probabilistic Approach to Extreme Statistics of Brownian Escape Times in Dimensions 1, 2, and 3. *J. Nonlinear Sci.* 30:1207–1227.

36. Lindsay, A. E., A. J. Bernoff, and A. Navarro Hernández. 2023. Short-Time Diffusive Fluxes over Membrane Receptors Yields the Direction of a Signalling Source. *R. Soc. Open Sci.* 10:221619.

37. van Zon, J. S., and P. R. ten Wolde. 2005. Green's-Function Reaction Dynamics: A Particle-Based Approach for Simulating Biochemical Networks in Time and Space. *J. Chem. Phys.* 123:234910.

38. van Zon, J. S., and P. R. ten Wolde. 2005. Simulating Biochemical Networks at the Particle Level and in Time and Space: Green's Function Reaction Dynamics. *Phys. Rev. Lett.* 94:128103.

39. Bernstein, D. 2005. Simulating Mesoscopic Reaction-Diffusion Systems Using the Gillespie Algorithm. *Phys. Rev.* 71:041103.

40. Smoluchowski, M. v. 1918. Versuch einer mathematischen Theorie der Koagulationskinetik kolloider Lösungen. *Zeitschrift für Physikalische Chemie.* 92U:129–168.

41. Andrews, S. S., and D. Bray. 2004. Stochastic Simulation of Chemical Reactions with Spatial Resolution and Single Molecule Detail. *Phys. Biol.* 1:137–151.

42. Kerr, R. A., T. M. Bartol, …, J. R. Stiles. 2008. Fast Monte Carlo Simulation Methods for Biological Reaction-Diffusion Systems in Solution and on Surfaces. *SIAM J. Sci. Comput.* 30:3126–3149.

43. Klann, M., and H. Koeppl. 2012. Spatial Simulations in Systems Biology: From Molecules to Cells. *Int. J. Mol. Sci.* 13:7798–7827.

44. Schöneberg, J., A. Ullrich, and F. Noé. 2014. Simulation Tools for Particle-Based Reaction-Diffusion Dynamics in Continuous Space. *BMC Biophys.* 7:11.

45. Agbanusi, I. C., and S. A. Isaacson. 2014. A Comparison of Bimolecular Reaction Models for Stochastic Reaction-Diffusion Systems. *Bull. Math. Biol.* 76:922–946.

46. Johnson, M. E., A. Chen, …, A. M. Uhrmacher. 2021. Quantifying the Roles of Space and Stochasticity in Computer Simulations for Cell Biology and Cellular Biochemistry. *Mol. Biol. Cell.* 32:186–210.

47. Young, M. E., P. A. Carroad, and R. L. Bell. 1980. Estimation of Diffusion Coefficients of Proteins. *Biotechnol. Bioeng.* 22:947–955.

48. Huang, Z., K. H. Pearce, and N. L. Thompson. 1994. Translational Diffusion of Bovine Prothrombin Fragment 1 Weakly Bound to Supported Planar Membranes: Measurement by Total Internal Reflection with Fluorescence Pattern Photobleaching Recovery. *Biophys. J.* 67:1754–1766.

49. Olson, S. T., I. Björk, and J. D. Shore. 1993. [30] Kinetic Characterization of Heparin-Catalyzed and Uncatalyzed Inhibition of Blood Coagulation Proteinases by Antithrombin. *In* Methods in Enzymology, Academic Press, Volume 222 of *Proteolytic Enzymes in Coagulation, Fibrinolysis, and Complement Activation Part A: Mammalian Blood Coagulation Factors and Inhibitors*, pp. 525–559.

50. Conard, J., F. Brosstad, …, U. Abildgaard. 1983. Molar Antithrombin Concentration in Normal Human Plasma. *Haemostasis.* 13:363–368.

51. Nesheim, M. E., R. P. Tracy, …, K. G. Mann. 1992. Mathematical Simulation of Prothrombinase. *Methods in Enzymology.* 215:316–328.

52. Weiss, H. J. 1975. Platelet Physiology and Abnormalities of Platelet Function. *N. Engl. J. Med.* 293:531–541.

53. Montgomery, D., F. Municchi, and K. Leiderman. 2023. clotFoam: An open-source framework to simulate blood clot formation under arterial flow. *SoftwareX.* 23:101483. https://www.sciencedirect.com/science/article/pii/S2352711023001796.

54. Andrews, S. S., N. J. Addy, …, A. P. Arkin. 2010. Detailed Simulations of Cell Biology with Smoldyn 2.1. *PLoS Comput. Biol.* 6:e1000705.

55. Andrews, S. S. 2012. Spatial and Stochastic Cellular Modeling with the Smoldyn Simulator. *Methods Mol. Biol.* 804:519–542.

56. Yogurtcu, O. N., and M. E. Johnson. 2015. Theory of Bi-Molecular Association Dynamics in 2D for Accurate Model and Experimental Parameterization of Binding Rates. *J. Chem. Phys.* 143:084117.

57. Chen, A., T. Wessler, …, M. G. Forest. 2022. Modeling Insights into SARS-CoV-2 Respiratory Tract Infections Prior to Immune Protection. *Biophys. J.* 121:1619–1631.

58. Pablo, M., S. A. Ramirez, and T. C. Elston. 2018. Particle-Based Simulations of Polarity Establishment Reveal Stochastic Promotion of Turing Pattern Formation. *PLoS Comput. Biol.* 14:e1006016.

59. Rosing, J., G. Tans, …, H. C. Hemker. 1980. The Role of Phospholipids and Factor Va in the Prothrombinase Complex. *J. Biol. Chem.* 255:274–283.

60. Connor, J., C. C. Pak, and A. J. Schroit. 1994. Exposure of Phosphatidylserine in the Outer Leaflet of Human Red Blood Cells. Relationship to Cell Density, Cell Age, and Clearance by Mononuclear Cells. *J. Biol. Chem.* 269:2399–2404.

61. Bevers, E. M., P. Comfurius, and R. F. Zwaal. 1991. Platelet Procoagulant Activity: Physiological Significance and Mechanisms of Exposure. *Blood Rev.* 5:146–154.

62. Abaeva, A. A., M. Canault, …, M. A. Panteleev. 2013. Procoagulant Platelets Form an α-Granule Protein-covered "Cap" on Their Surface That Promotes Their Attachment to Aggregates*. *J. Biol. Chem.* 288:29621–29632.

63. Podoplelova, N. A., A. N. Sveshnikova, …, M. A. Panteleev. 2016. Coagulation Factors Bound to Procoagulant Platelets Concentrate in Cap Structures to Promote Clotting. *Blood.* 128:1745–1755.

64. Storrie, B. 2016. A Tip of the Cap to Procoagulant Platelets. *Blood.* 128:1668–1669.

65. Van Beers, E. J., M. C. L. Schaap, …, B. J. Biemond. 2009. Circulating Erythrocyte-Derived Microparticles Are Associated with Coagulation Activation in Sickle Cell Disease. *Haematologica.* 94:1513–1519.

66. Setty, B. N. Y., S. Kulkarni, and M. J. Stuart. 2002. Role of Erythrocyte Phosphatidylserine in Sickle Red Cell–Endothelial Adhesion. *Blood.* 99:1564–1571.

67. Thomas, G. M., A. Brill, …, D. D. Wagner. 2015. Tissue Factor Expressed by Circulating Cancer Cell-derived Microparticles Drastically Increases the Incidence of Deep Vein Thrombosis in Mice. *J. Thromb. Haemost.* 13:1310–1319.

68. Zwicker, J. I., B. C. Furie, and B. Furie. 2007. Cancer-Associated Thrombosis. *Crit. Rev. Oncol. Hematol.* 62:126–136.

69. Bobone, S., M. Hilsch, …, S. Chiantia. 2017. Phosphatidylserine Lateral Organization Influences the Interaction of Influenza Virus Matrix Protein 1 with Lipid Membranes. *J. Virol.* 91:e00267-17. https://doi.org/10.1128/jvi.00267–17.

70. Ishii, H., T. Mori, …, Y. Nakanishi. 2005. Distinct Localization of Lipid Rafts and Externalized Phosphatidylserine at the Surface of Apoptotic Cells. *Biochem. Biophys. Res. Commun.* 327:94–99.

71. PIKE, L. J. 2004. Lipid Rafts: Heterogeneity on the High Seas. *Biochem. J.* 378:281–292.

72. Bodin, S., H. Tronchère, and B. Payrastre. 2003. Lipid Rafts Are Critical Membrane Domains in Blood Platelet Activation Processes. *Biochim. Biophys. Acta Biomembr.* 1610:247–257.

73. Luc, N. F., N. Rohner, …, A. Sen Gupta. 2022. Bioinspired Artificial Platelets: Past, Present and Future. *Platelets.* 33:35–47.

Biophysical Journal, Volume *125*

Supplemental information

Continuum and discrete modeling of binding-site distribution-mediated reactions on lipid surfaces

Han Cao, Anirban Sen Gupta, and Karin Leiderman

Continuum and discrete modeling of binding-site distribution-mediated reactions on lipid surface

Authors: Han Cao, Anirban Sen Gupta, Karin Leiderman

SUPPLEMENTAL INFORMATION

PDE numerical scheme convergence analysis

To solve the coupled bulk–surface PDE model, we employed an operator-splitting time-stepping scheme. Here, we provide a simple convergence analysis to assess the numerical behavior of this scheme.

Because the full model involves multiple coupled species and nonlinear reaction terms and does not admit an analytical solution, we instead considered a reduced test problem that captures the essential bulk–surface coupling mechanism: a single chemical species undergoing diffusion in the bulk and reversible binding on the surface.

Specifically, we consider the following system:

$$\frac{\partial u}{\partial t} = D\Delta u, \tag{1}$$

$$\frac{\partial u^{\mathrm{m}}}{\partial t} = D^{m}\Delta_{\Gamma}u^{\mathrm{m}} + k_{\mathrm{on}}\, u\,(b_{\mathrm{total}} - u^{\mathrm{m}}) - k_{\mathrm{off}}\, u^{\mathrm{m}}, \tag{2}$$

$$D\,\frac{\partial u}{\partial \mathbf{n}} = k_{\mathrm{off}}\, u^{\mathrm{m}} - k_{\mathrm{on}}\, u\,(b_{\mathrm{total}} - u^{\mathrm{m}}), \tag{3}$$

where u denotes the solution-phase concentration and u^{m} denotes the surface-bound density. Here, b_{total} represents the total binding-site density on the surface, which is assumed to be spatially uniform. This configuration corresponds to binding patch distribution Pattern 1 shown in Fig. 4A of the main text, in which binding sites are uniformly distributed along the membrane.

Temporal convergence

We first examined the temporal convergence behavior of the operator-splitting scheme by systematically refining the time step size dt while keeping the spatial discretization fixed. For each choice of dt, we computed the spatially averaged bulk concentration $u(t)$, the spatially averaged surface-bound density $u^{\mathrm{m}}(t)$, and the total mass $u(t) + u^{\mathrm{m}}(t)$ with appropriate dimensional scaling. All quantities were normalized by their initial (or maximal) values.

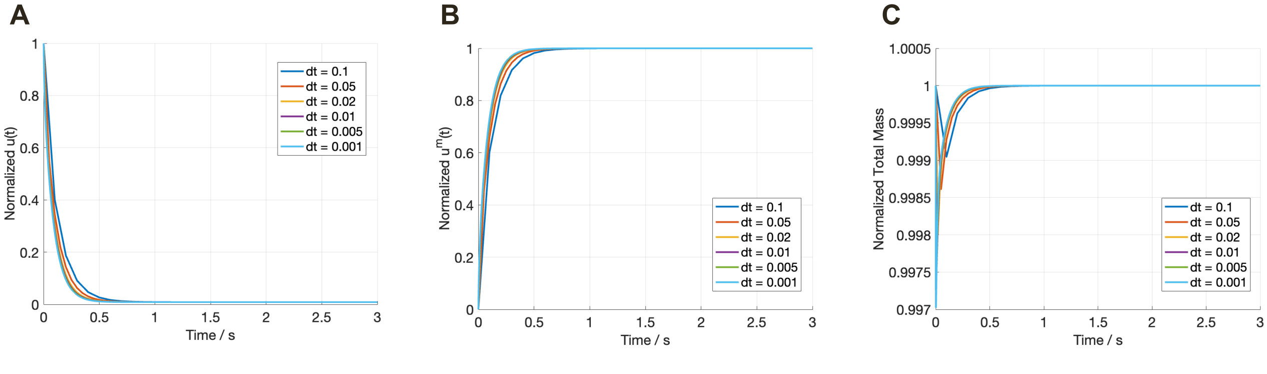

Figure S1: Temporal convergence of the operator-splitting scheme for the reduced bulk–surface binding model. (A) Normalized spatially averaged bulk concentration $u(t)$. (B) Normalized spatially averaged surface-bound concentration $u^{\mathrm{m}}(t)$. (C) Normalized total mass $u(t) + u^{\mathrm{m}}(t)$, with proper dimensional scaling. Results are shown for decreasing time step sizes dt.

Figure S1 shows the resulting time courses for decreasing values of dt. Panel A displays the normalized bulk concentration $u(t)$, Panel B shows the normalized surface-bound concentration $u^{\mathrm{m}}(t)$, and Panel C shows the normalized total mass. As the time step is refined, the solution trajectories converge toward a common curve for both the bulk and surface concentrations, indicating consistent temporal convergence of the scheme.

A small transient deviation from perfect mass conservation is observed at early times. This deviation is a numerical artifact of the operator-splitting treatment of the bulk–surface coupling. Because diffusion in the bulk and binding/unbinding reactions

on the surface are advanced sequentially, the bulk–surface mass exchange is not enforced in a fully coupled manner within each time step. As a result, small splitting errors in the total mass can be observed at early times. These deviations rapidly diminish as the simulation proceeds, and the total mass approaches a near-constant value at longer times.

Spatial convergence

We next examined the sensitivity of the numerical solution to the spatial discretization in the membrane-normal direction by refining the grid spacing dy, while keeping the time step fixed. Because the system is homogeneous in the x-direction for this test problem, only refinement in the y-direction was considered. Figure S2 shows the normalized solution-phase concentration u(t), surface-bound density $u^{\mathrm{m}}(t)$, and the normalized total mass for several values of dy.

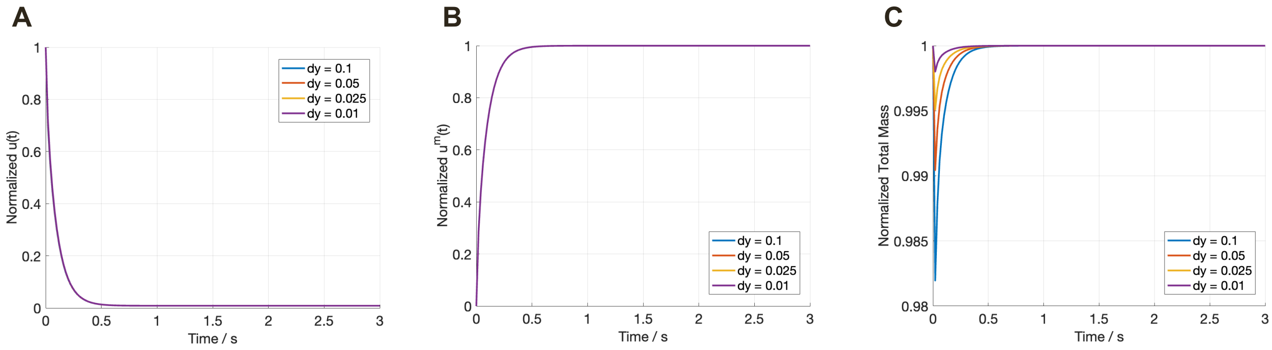

Figure S2: Spatial convergence of the operator-splitting scheme for the reduced bulk–surface binding model. (A) Normalized spatially averaged bulk concentration $u(t)$. (B) Normalized spatially averaged surface-bound concentration $u^{\mathrm{m}}(t)$. (C) Normalized total mass $u(t) + u^{\mathrm{m}}(t)$, with proper dimensional scaling. Results are shown for decreasing decreasing spatial grid spacing dy.

Across all tested spatial resolutions, the solution-phase and surface-bound concentration curves are nearly indistinguishable. This indicates that the temporal evolution of both u(t) and $u^{\mathrm{m}}(t)$ is well resolved even on relatively coarse spatial grids, and that further refinement in dy does not lead to visually discernible changes in these quantities.

In contrast, the small numerical artifact observed in the total mass conservation becomes progressively smaller as dy is refined. This behavior is consistent with discretization error in the bulk–surface coupling at the membrane boundary. As the spatial resolution increases, the discrete approximation of the normal flux and surface reaction terms improves, leading to better mass balance between the bulk and membrane compartments.

Temporal convergence of the full PDE model

We next examined the temporal convergence behavior of the full PDE model presented in the main text by repeating simulations using multiple time step sizes (dt = 0.01, 0.005, and 0.0025 s). Binding patch pattern was again set to be Pattern 1 shown in Fig. 4A from the main text.

As shown in Fig. S3, the temporal evolution of the solution-phase enzyme concentration $\mathrm{E}_1(t)$ and the membrane-bound enzyme density $\mathrm{E}_1^{\mathrm{m}}(t)$ is nearly identical across all tested values of dt, including both the peak magnitude and the overall time course. Here, $\mathrm{E}_1(t)$ denotes the spatially averaged enzyme concentration over the solution domain, and $\mathrm{E}_1^{\mathrm{m}}(t)$ denotes the spatially averaged enzyme density on the membrane.

These results indicate that, over the range of time step sizes considered, the numerical solution of the full PDE model exhibits weak sensitivity to temporal discretization, and that the time step used in the main simulations is sufficient to resolve the relevant dynamics.

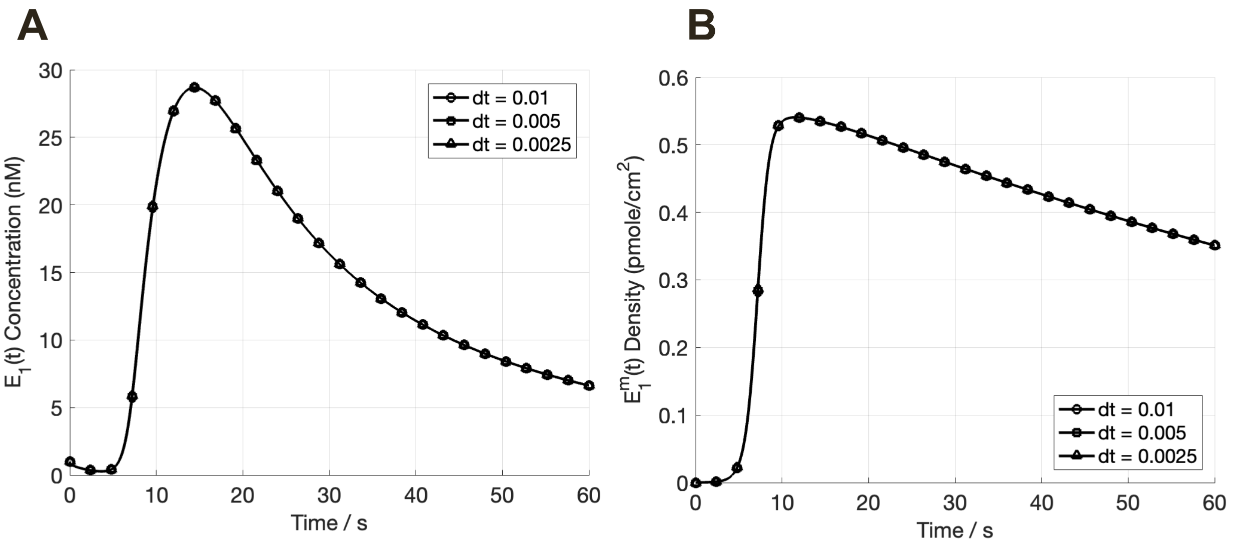

Figure S3: Temporal convergence behavior of the full PDE model presented in the main text. Results are shown for dt = 0.01, 0.005, and 0.0025 s. (A) Spatially averaged solution-phase enzyme concentration $E_1(t)$. (B) Spatially averaged membrane-bound enzyme density $E_1^m(t)$.

Taken together, these results demonstrate that the operator-splitting scheme employed here yields stable and convergent solution trajectories for the quantities of interest. Although the splitting approach introduces small transient mass-balance errors due to the sequential treatment of bulk diffusion and surface reactions, these artifacts are controlled and do not materially influence the qualitative or quantitative conclusions drawn from the simulations.

Vesicle geometry has little effect on product generation in 3D particle-based simulations

To enable precise control over binding patch geometry, we approximate the synthetic platelet as a cube, as Smoldyn currently provides more flexibility in defining surface regions on polyhedral geometries than on curved ones. Moreover, defining patches using triangular panels on a triangulated sphere introduces challenges: discretization results in panels of varying size and shape, making it difficult to uniformly control patch dimensions and spatial distribution.

To test the impact of synthetic platelet geometry, we performed simulations using three geometries with different levels of discretization: a perfect sphere, a cube, and a triangulated sphere composed of 80 triangular panels (see Fig. S4). The same reaction system and parameter values as described in the main study were used; however, in this analysis, binding site particles were allowed to diffuse freely across the entire platelet surface. If geometry played a significant role, such a setup would amplify any differences. Our results in Fig. S5, however, showed negligible differences in reaction outcomes across the three geometries, suggesting that platelet geometry has minimal influence under these conditions.

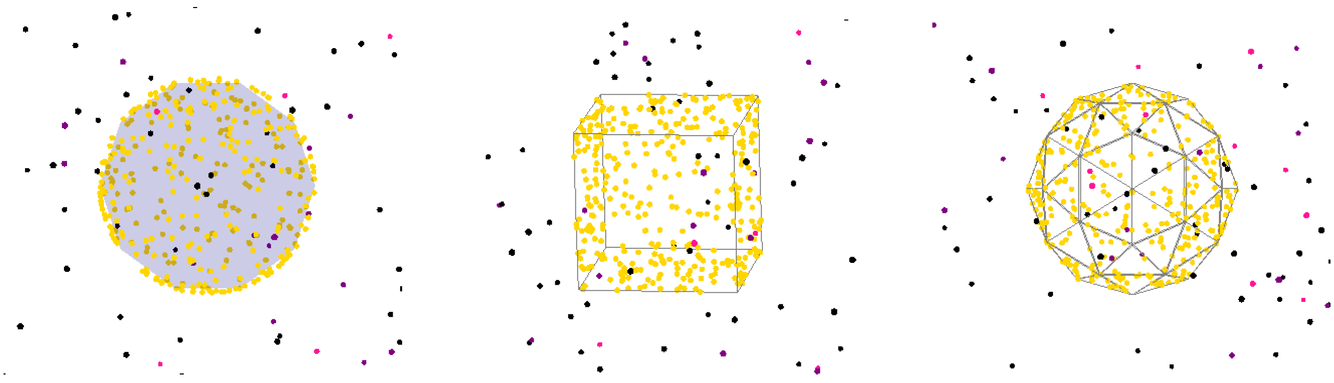

Figure S4: Snapshots of vesicles with different surface geometries. Yellow particles on the platelet surface represent binding site particles. (A) A vesicle with spherical surface. (B) A vesicle with cubic surface. (C) A vesicle with triangulated surface.

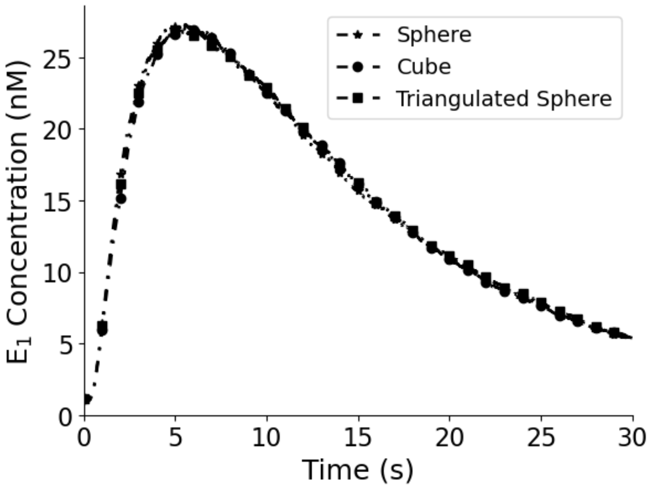

Figure S5: Enzyme generation curve for synthetic platelets of different geometries: a perfect sphere, a cube, and a triangulated sphere with 80 triangular panels.

Calculations

Platelet size and solution domain in PDE model

Assuming the surface of an activated platelet is a perfect sphere with radius

$$r_{\text{platelet}} = 2\mu\text{m},$$

then a cubic solution domain of side length

$$L_{\text{solution}} = 16\ \mu\text{m}$$

containing one platelet will yield a platelet concentration of

$$[\text{Platelet}] = 1/V_{\text{solution}} = 1/L^3_{\text{solution}} = 2.44 \times 10^5/\text{mm}^3,$$

which closely matches the physiological platelet concentration of approximately $2.5 \times 10^5/\text{mm}^3$. Placing the platelet at the center of the solution domain implies that the shortest distance from the platelet surface to the boundary of the solution domain is 6 μm, which is the value of $y_{\max}$ used in the PDE simulation (see Fig. S6).

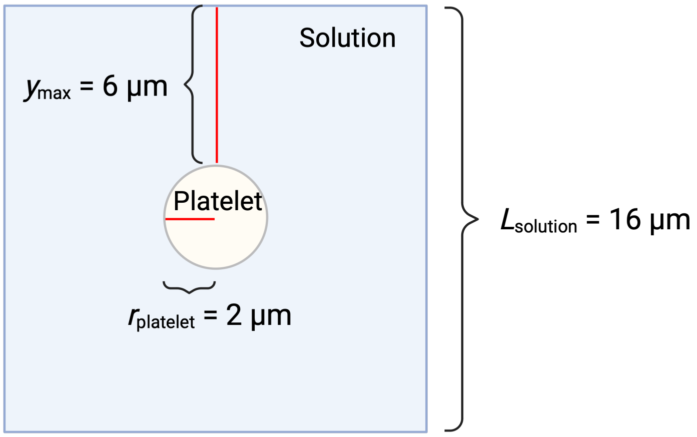

Figure S6: Schematic of a 2D cross-section of the platelet and its surrounding solution domain

Binding site density

We assumed each surface-bound protein occupies a circular area with a diameter of 5nm, giving a surface area of approximately

$$s_{\text{protein}} = (2.5\text{nm})^2\pi = 6.25\pi \text{ nm}^2.$$

This leads to an estimated maximum binding site density of $p_{\max} = 1/(6.25\pi\text{nm}^2) \approx 8.45$ pmole/cm^2. In the PDE simulations, we used a slightly larger value of 10 pmole/cm^2.

Lipid vesicle size and domain in 3D particle-based model

We assumed the lipid vesicle has the radius of

$$r_{\text{PPN}} = 75 \text{ nm}$$

as in (1). Then, the surface area of the lipid vesicle is

$$s_{\text{PPN}} = 4\pi r_{\text{PPN}}^2 = 0.0225\pi \ \mu\text{m}^2.$$

If the lipid vesicle is approximated as a cube with the same total surface area, the corresponding side length is

$$l_{\text{PPN}} = \sqrt{s_{\text{PPN}}/6} \approx 0.10854 \ \mu\text{m}.$$

To determine the appropriate solution domain size for a particle-based simulation, we matched the surface area-to-volume ratio to that of a real platelet. A single physiological platelet has the same surface area as approximately 711 synthetic platelets:

$$\frac{s_{\text{platelet}}}{s_{\text{PPN}}} = \frac{4\pi r_{\text{platelet}}^2}{4\pi r_{\text{PPN}}^2} \approx 711.$$

Thus, to maintain equivalent surface area per unit volume, the solution domain for one synthetic platelet should be

$$v_{\text{solution}} = \frac{V_{\text{solution}}}{711},$$

yielding a cubic domain of side length

$$l_{\text{solution}} = \sqrt[3]{v_{\text{solution}}} \approx 1.8 \ \mu\text{m}.$$

Number of binding site particles in the particle-based model

Binding site particles

We assumed that 10% of the lipids on the synthetic platelet surface effectively contribute as binding sites. Accordingly, 10% of the synthetic platelet surface is designated as binding patches. To estimate the number of binding site particles per synthetic platelet, we divided the total area of the binding patches by the surface area occupied by a single protein:

$$\# \text{ of BS} = \frac{s_{\text{PPN}} \times 10\%}{s_{\text{protein}}} = 360.$$

Enzyme and zymogen particles

If the enzyme concentrations are set to $[E_1] = [E_2] = 1$ nM, then the number of E_1 and E_2 molecules in the simulation domain can be estimated by multiplying the concentration by the solution volume v_{solution}

$$\# \text{ of } E_1(E_2) = 1 \text{ nM} \times v_{\text{solution}} \approx 3.51 \approx 4.$$

Since molecule counts must be integers in particle-based simulations, we initialize the system with 4 molecules each of E_1 and E_2 in the solution.

For the initial counts of zymogens, we use 400 molecules each of Z_1 and Z_2 in the solution.

Inhibitor particles

To ensure that all enzymes generated during the simulation can eventually be inhibited, we set the initial number of inhibitor molecules to 1000.

Stochastic effect of enzyme generation in 3D particle-based simulations

In some of our 3D particle-based simulations, enzyme inhibition occurs before amplification can begin, completely preventing the initiation of product generation—an inherently probabilistic outcome that cannot be captured by deterministic models.

In Fig.S7(A), we illustrate this effect using the case of a single binding patch as an example. Here, E_1 concentration trajectories from 200 simulation runs are shown, where the red line highlights runs in which no initiation occurs and the enzyme count decays to zero before product generation begins. Fig.S7(B) further summarizes the number of non-initiated simulations observed under each binding site distribution.

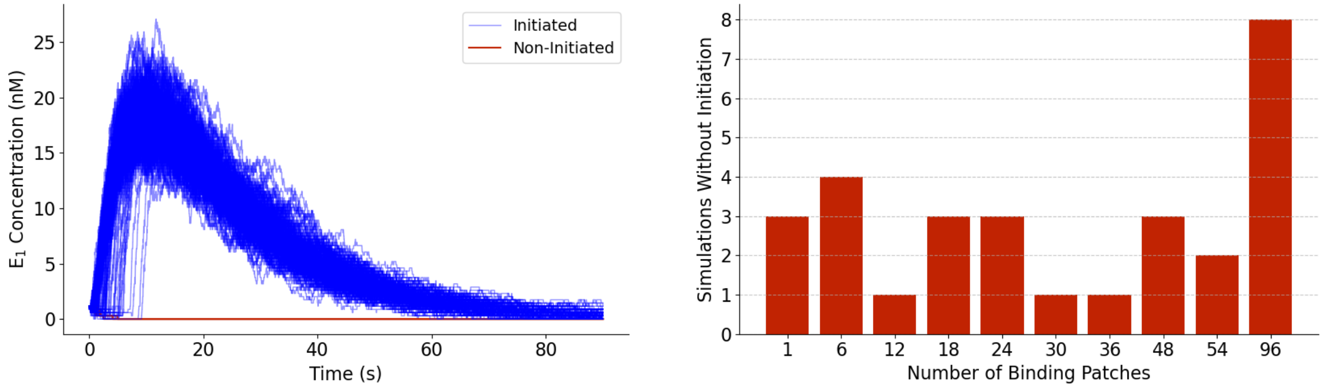

Figure S7: (A) Simulation trajectories for all runs with one binding patch. Trajectories where reactions were successfully initiated are shown in blue, while those with no reaction initiation are shown in red. This highlights the stochastic variability inherent at the molecular scale and low molecule counts, which deterministic models cannot capture. (B) Number of simulations with no reaction initiation (non-initiated) across different numbers of binding patches. The count reflects the stochastic failure of reaction initiation in particle-based simulations.

REFERENCES

1. Sekhon, U. D. S., K. Swingle, A. Girish, N. Luc, M. de la Fuente, J. Alvikas, S. Haldeman, A. Hassoune, K. Shah, Y. Kim, S. Eppell, J. Capadona, A. Shoffstall, M. D. Neal, W. Li, M. Nieman, and A. Sen Gupta, 2022. Platelet-Mimicking Procoagulant Nanoparticles Augment Hemostasis in Animal Models of Bleeding. *Science Translational Medicine* 14:eabb8975.
